# Small Singlet–Triplet Gap Terpolymer Donor with a Simple Pt Complex Enables Organic Solar Cells with Low Energy Loss and Over 19.2% Efficiency

**DOI:** 10.1002/advs.202410154

**Published:** 2025-02-06

**Authors:** Dou Luo, Lifu Zhang, Lanqing Li, Tingting Dai, Erjun Zhou, Mao Quan, Hongyang Zhang, Aung Ko Ko Kyaw, Wai‐Yeung Wong

**Affiliations:** ^1^ Department of Applied Biology and Chemical Technology and Research Institute for Smart Energy The Hong Kong Polytechnic University Hung Hom Hong Kong 999077 P. R. China; ^2^ The Hong Kong Polytechnic University Shenzhen Research Institute Shenzhen 518057 P. R. China; ^3^ Institute of Advanced Scientific Research (iASR)/Key Lab of Fluorine and Silicon for Energy Materials and Chemistry of Ministry of Education Jiangxi Normal University Nanchang 330022 P. R. China; ^4^ School of Pharmacy and Food Engineering Wuyi University Jiangmen 529020 P. R. China; ^5^ National Center for Nanoscience and Technology Beijing 100190 P. R. China; ^6^ College of Materials Science and Engineering Shenzhen University Xueyuan Blvd 1066 Shenzhen 518055 P. R. China; ^7^ Guangdong University Key Laboratory for Advanced Quantum Dot Displays and Lighting Department of Electronic & Electrical Engineering Southern University of Science and Technology Shenzhen 518055 P. R. China

**Keywords:** morphology, non‐radiative recombination, organic solar cells, platinum complex, triplet state

## Abstract

Suppressing the non‐radiative loss in the organic solar cells (OSCs) through molecular design remains a significant challenge. Typically, triplet state of organic semiconductors is lower than the charge transfer (CT) state, contributing to substantial non‐radiative loss via the triplet state. Herein, a set of terpolymers is prepared by introducing a simple Pt complex block into the PM6 polymer backbone. These metalated terpolymers exhibit high triplet energy (*E*_T1_) and small singlet–triplet energy gap (∆*E*_ST_), facilitating fast intersystem crossing (ISC) process to generate triplet excitons. Consequently, the metalated terpolymers show enhanced exciton lifetime and diffusion length, and most importantly, effectively suppress the non‐radiative recombination via terminal triplet loss channels. Moreover, the Pt complex modifies the molecular aggregation of the polymer, hence optimizing the morphology of the active blends. The PM6‐Pt1:L8‐BO devices achieve a champion power conversion efficiency (PCE) of 18.54% (certified as 18.32%), the highest reported for metalated terpolymers to date. The PCE is further increased to a record high 19.24% in the PM6‐Pt1:PM6:L8‐BO (0.8:0.2:1.2, wt/wt/wt) ternary devices. Overall, this work provides a feasible approach to designing terpolymers with high *E*_T1_, thereby reducing non‐radiative loss in the OSCs.

## Graphical abstract

A set of terpolymers is prepared by introducing a simple Pt complex block into the PM6 polymer backbone. These metalated terpolymers exhibit small singlet–triplet energy gap, facilitating fast intersystem crossing process to generate triplet excitons. The PM6‐Pt1:L8‐BO and PM6‐Pt1:PM6:L8‐BO devices achieve a champion power conversion efficiency (PCE) of 18.54% (certified as 18.32%) and 19.24%, respectively.

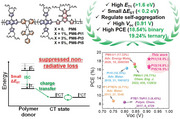

## 1 Introduction

Organic solar cells (OSCs) have attracted great research attention owing to their low cost, light weight, and large‐scale flexibility.^[^ ^1^, ^2^, ^3^, ^4^ ^]^ Notably, the development of advanced fused‐ring small molecular non‐fullerene acceptors (NFAs) has enabled the power conversion efficiencies (PCEs) to exceed 19% in single‐junction OSCs.^[^ ^5^, ^6^, ^7^, ^8^, ^9^ ^]^ At present, the key factor limiting the improvement of PCEs is the relatively large energy loss (*E*_loss_), which severely restricts the open‐circuit voltage (*V*_oc_).^[^ ^10^ ^]^ The working mechanism of OSCs can be simply represented as four steps: light absorption and exciton generation, exciton diffusion, charge separation, and charge transport and collection.^[^ ^11^ ^]^ Thus, the PCE of OSCs depends on the efficiency of each step. Specially, one of the main challenges in reducing *E*_loss_ is to suppress exciton recombination of charge pairs formed by charge transfer (CT) at the donor (D)–acceptor (A) mixed phase.^[^ ^12^, ^13^ ^]^ *E*_loss_ can be primarily attributed to the radiative recombination and non‐radiative recombination, with the former being difficult to reduce further due to the intrinsic properties of NFAs.^[^ ^14^ ^]^ Consequently, non‐radiative recombination predominantly determines the *E*_loss_ in OSCs due to the significant energetic disorder of organic semiconductors.^[^ ^15^ ^]^ In this regard, to further improve the performance of OSCs, it is essential to develop effective strategies to minimize the non‐radiative recombination loss.

Several factors, including triplet exciton recombination, morphology defects, and energetic traps, influence the non‐radiative recombination loss.^[^ ^16^ ^]^ Extending the exciton diffusion length (*L*_D_) can reduce the likelihood of exciton recombination before diffusing to the D/A interface, thereby reducing the non‐radiative recombination loss. For example, Zhan et al. reported that incorporating a highly emissive *trans*‐bis(dimesitylboron)stilbene (BBS) solid additive improved the charge‐carrier diffusion length from 65.4 ± 3.1 nm for PM6:Y6 to 68.2 ± 3.9 nm for PM6:Y6:BBS films, resulting in reduced charge recombination and energy loss.^[^ ^17^ ^]^ This long diffusion length led to a higher PCE of 17.6% for the PM6:Y6:BBS devices compared to 16.2% for the devices without BBS. Similarly, Zhao et al. proposed a method to extend *L*_D_ from 7.9 to 10.7 nm by deuterating the acceptor, which reduced vibrational frequency and suppress exciton‐vibration coupling, thereby decreasing the non‐radiative decay rate.^[^ ^18^ ^]^ The resulting L8‐BO‐OD‐based pseudo‐planar heterojunction OSCs achieved an impressive PCE of 19.3%, outperforming the control devices (17.6%). In addition, it has been observed that the photogenerated triplet charge transfer state (^3^CT) can easily relax to the triplet excited state when the energy level of ^3^CT is higher than the triplet energy (*E*_T1_) of donor materials, resulting in a considerable non‐radiative recombination loss.^[^ ^12^, ^19^ ^]^ Therefore, to reduce non‐radiative recombination loss via triplet loss channel, one promising strategy is to elevate the *E*_T1_ for achieving a small singlet–triplet energy gap (∆*E*_ST_). B─N bond was first introduced into the polymer donors and acceptors by Duan et al. and they confirmed the small Δ*E*_ST_ and high *E*_T1_ in the related materials.^[^ ^20^, ^21^, ^22^, ^23^ ^]^ After this, Peng et al. reported high‐performance OSCs using triplet polymer donors featuring B─N bond and small Δ*E*_ST_.^[^ ^24^ ^]^ The resulting polymer PNB‐3 exhibited a boosted PCE compared to the benchmark polymer PM6 (17.92% vs 19.02%) with a lower *E*_loss_ (0.547 eV vs 0.533 eV). Moreover, incorporating heavy atoms into conjugated systems can enhance spin–orbit coupling (SOC), facilitating intersystem crossing (ISC) from the lowest singlet state (S_1_) to T_1_, to generate triplet excitons. Triplet excitons generally have longer lifetimes than single excitons, allowing them to travel further in the complex mixed phase. Huang et al. introduced iridium into the backbone of PTB7, significantly improving the efficiency of OSCs by over 40%, resulting in an efficiency of 8.71%.^[^ ^25^ ^]^ Min et al. added a 1% iridium complex, (dfppy)2IrdbmBr, to the polymer conjugated backbone of PM6.^[^ ^26^ ^]^ The PM6‐Ir1:Y6‐based devices exhibited an enhanced PCE of 17.24%, attributed to suppressed charge recombination and optimized morphology. In this regard, leveraging the advantage of heavy metal atom to develop new triplet polymers can lower the Δ*E*_ST_ and further reduce the non‐radiative recombination loss, thus leading to OSCs of higher performance.

**Figure 1:**
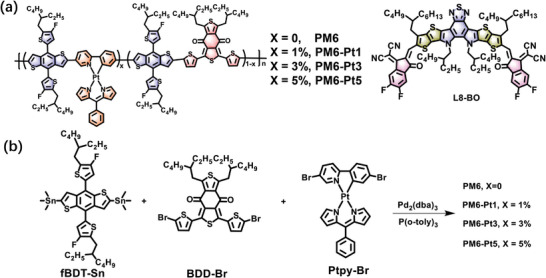
a) Chemical structures of the terpolymer donors and L8‐BO acceptor. b) Synthetic procedures of the terpolymers.

In this study, we present new terpolymers created by incorporating different concentrations of a simple platinum (Pt) complex (0, 1, 3, and 5 mol%; Ptpy) into the polymer conjugated backbone of PM6 (**Figure** **1**a). Ptpy fragment consists of dipyrrin, which can act as a monoanionic chelate and form complexes with Pt center. Moreover, dipyrrin complexes can generate strong luminescence, but have been less investigated. Besides, Ptpy could exhibit microsecond triplet lifetimes and high absorption coefficient in the visible light range. Considering the properties of Ptpy monomer, we selected Ptpy as the third component to prepare metalated terpolymers. Single crystal analysis revealed strong π–π stacking and C─H**^…^** π interactions among the Ptpy monomer. These metalated terpolymers achieved higher *E*_T1_ (≈1.64 eV) and smaller Δ*E*_ST_ (≈0.19 eV) compared to the control PM6 (*E*_T1_ < 1.23 eV and Δ*E*_ST_ > 0.6 eV), resulting in a rapid ISC process to produce triplet excitons. The rate constants for ISC (*k*_ISC_) from the lowest S_1_ to T_1_ were determined through both experimental tests and theoretical calculations. The as‐synthesized metalated terpolymers exhibited longer exciton lifetime and *L*_D_ compared to the benchmark polymer PM6. Importantly, non‐radiative recombination via the terminal triplet loss channels was significantly suppressed. Introducing the Ptpy monomer also regulated polymer self‐aggregation, hence achieving optimal morphology. Among the metalated polymers, Pt1 with 1 mol% Ptpy monomer achieved the highest PCE of 18.54% (certified as 18.32%), with a high *V*_oc_ of 0.91 V and a low non‐radiative recombination loss of 0.219 eV when paired with L8‐BO acceptor. This performance surpasses that of PM6:L8‐BO devices, which have a PCE of 18.1%, a *V*_oc_ of 0.88 V, and a non‐radiative recombination loss of 0.234 eV. To the best of our knowledge, this is the highest PCE reported for OSCs using metalated terpolymers as electron donors. Additionally, ternary devices based on PM6‐Pt1:PM6:L8‐BO (0.8:0.2:1.2, wt/wt/wt) achieved a record high PCE of 19.24%. Our results indicate that incorporating simple heavy metal complexes into benchmark polymers can generate terpolymers with small Δ*E*_ST_ and low *E*_loss_, thereby reducing charge recombination and enhancing the efficiency of OSCs.

## 2 Results and Discussion

### 2.1 Photoelectric Properties of Monomers and Polymers

The synthetic routes of the terpolymers are shown in Figure 1b. The targeted conjugated polymers PM6‐Pt1, PM6‐Pt3, and PM6‐Pt5, with varying concentrations of Pt complex, were synthesized through the Stille‐coupling polymerization of organotin monomer (fBDT‐Sn) with brominated monomers of BDD‐Br and Ptpy‐Br. The dibrominated monomer Ptpy‐Br was synthesized with high yield (Scheme S1, Supporting Information). After the same reaction time, the number‐average molecular weights (*M*_n_s) of the polymers were determined to be 20.1, 18.6, and 16.7 kDa, with polydispersity indexes (PDIs) of 2.52, 2.95, and 2.77 for PM6‐Pt1, PM6‐Pt3, and PM6‐Pt5, respectively, as measured by high‐temperature gel permeation chromatography, suggesting that the introduction of more Pt complex has a slight impact on the molecular weight (**Table** **1**; Figure S1, Supporting Information). Furthermore, the Pt content was detected by inductively coupled plasma optical emission spectrometer (ICP‐OES). The Pt contents for PM6‐Pt1, PM6‐Pt3, and PM6‐Pt5 were 1.601, 4.834, and 8.026 µg mg^−1^, respectively (Table S1, Supporting Information), implying the presence of ≈1%, 3%, and 5% of Ptpy monomer in the corresponding polymers.

**Table 1:** Molecular weight, optical properties, and energy levels of polymer donors.

Polymer	*M*n	*Ð*_M_	λ_max_ ^a)^	λ_onset_ ^b)^	*E*_g_^opt^ ^c)^	HOMO^d)^	LUMO^e)^
	[kDa]		[nm]	[nm]	[eV]	[eV]	[eV]
PM6‐Pt1	20.1	2.52	601	685	1.81	−5.60	−3.79
PM6‐Pt3	18.6	2.95	611	685	1.81	−5.68	−3.87
PM6‐Pt5	16.7	2.77	601	685	1.81	−5.69	−3.88

^a)^ Chloroform solution;

^b)^ As cast film;

^c)^ Calculated from the onset of the absorption spectrum of the film. *E*_g_^opt^ = 1240/λ_onset_;

^d)^ Evaluated by CV measurements;

^e)^ Calculated by *E*_LUMO_ = *E*_g_^opt^ + *E*_HOMO_.

**Figure 2:**
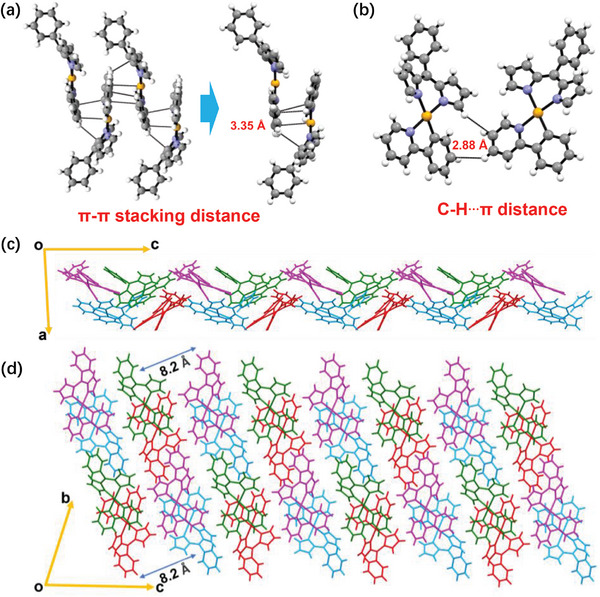
The single crystal of Ptpy, including a) π–π stacking distance and b) C─H**^…^** π distance. c) Crystal packing viewed along the b‐axis. d) Crystal packing viewed along the a‐axis.

To investigate the effect of introducing Pt complex on the molecular stacking of the terpolymers, the interactions among the Ptpy monomers were first explored. Single crystal analysis revealed that Ptpy exhibits a very short π–π stacking distance of 3.35 Å with a slip‐stacked head‐to‐tail packing model (**Figure** **2**a). This short π–π stacking distance should reinforce intermolecular interaction in the terpolymers. Furthermore, C─H**^…^** π interaction with a distance of 2.88 Å was observed between adjacent molecules (Figure 2b). This enhances the molecular interaction and weakens the vibrational coupling between molecules. The crystal packing viewed along the b‐axis and a‐axis is shown in Figure 2c,d. Ptpy shows a clear lamellar stacking feature in the crystal with a short d‐spacing of 8.2 Å. From the single crystal results of Pt complex, it is evident that the strong π–π stacking and C─H**^…^** π interactions of Ptpy will influence the intermolecular interactions in the terpolymers.

**Figure 3:**
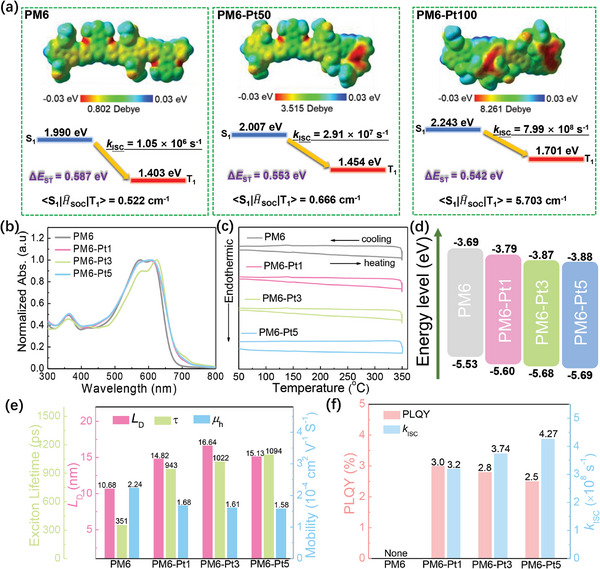
a) Electrostatic potential (ESP) and singlet, triplet energy level diagrams including *k*_ISC_ and SOC constants of PM6, PM6‐Pt50, and PM6‐Pt100. b) UV–vis absorption spectra and c) DSC curves of the polymers. d) Energy level diagram of the polymers. e) The *L*_D_, exciton lifetime, and hole mobility of the polymers. f) PLQY and the calculated *k*_ISC_ of the polymer films.

The impact of incorporating Ptpy monomer on the frontier energy levels was studied using theoretical method based on the density functional theory (DFT). By replacing the BDD unit with Ptpy in the polymer main chain, the HOMO/LUMO of three dimers PM6, PM6‐Pt50 (BDD:Ptpy = 1:1) and PM6‐Pt100 (BDD:Ptpy = 0:1) were calculated to be −5.11/−2.79, −5.23/−2.82, and −5.25/−2.66 eV, respectively (Figure S2a, Supporting Information). The deeper HOMO levels of the Pt complex‐based terpolymers suggest a potential for increased *V*_oc_ in the resulting OSCs. Besides, with increasing the Ptpy ratio, terpolymers (PM6‐Pt100) exhibited more separated frontier energy levels, which is expected to reduce the Δ*E*_ST_ and enhance the ISC process.^[^ ^8^ ^]^ Moreover, a small dihedral angle of 3.20° between the BDT and Ptpy units was observed in the PM6‐Pt50 polymer, while this angle increased to over 20° in the PM6‐Pt100 polymer, indicating that the introduction of the Pt complex monomer slightly influences the rigidity of the polymer backbone (Figure S2b, Supporting Information). According to the electrostatic potential (ESP) distribution, PM6‐Pt50 and PM6‐Pt100 polymers exhibited more negative potential on the Ptpy unit compared to PM6, suggesting more distinct ESP distributions and favorable charge delocalization for the Pt incorporated polymers (**Figure** **3**a). The dipole moments of the dimers significantly increased to 3.51 and 8.26 Debye for PM6‐Pt50 and PM6‐Pt100, respectively, indicating a stronger intermolecular charge transfer (ICT) effect in these polymers. The SOC constant (S_1_, T_1_) of PM6‐Pt1 (0.666 cm^−1^) was higher than that of PM6 (0.522 cm^−1^), and further significantly increased to 5.703 cm^−1^ for PM6‐Pt100. According to the perturbation theory, the increased SOC constants are beneficial for achieving high *k*_ISC_.^[^ ^27^, ^28^ ^]^ The *k*_ISC_ values were calculated to be 1.05 × 10^6^ s^−1^, 2.91 × 10^7^ s^−1^, and 7.99 × 10^8^ s^−1^ for PM6, PM6‐Pt50, and PM6‐Pt100, respectively, suggesting a more efficient ISC process with higher Pt complex ratios in the benchmark polymer. Furthermore, we also calculated the lowest singlet energy (*E*_S1_) and *E*_T1,_ as shown in Figure 3a. Both *E*_S1_ and *E*_T1_ were elevated with higher Pt complex ratios, while Δ*E*_ST_ decreased from 0.587 eV in PM6 to 0.542 eV in PM6‐Pt100, indicating that introducing Pt complex monomer into PM6 reduces Δ*E*_ST_. These results indicate that more triplet excitons with longer lifetimes can be generated through efficient ISC and small Δ*E*_ST_. The triplet excitons favor efficient exciton diffusion and contribute to charge transport in the devices.

The ultraviolet‐visible (UV–vis) absorption spectra of polymers in chloroform solution are shown in Figure S3 (Supporting Information). PM6‐Pt1 and PM6‐Pt5 exhibited similar absorption profiles to PM6. However, PM6‐Pt3 displayed a more pronounced vibronic 0–0 peak (*λ*_0–0_) and redshifted absorption compared to other polymers, likely due to enhanced ICT caused by the Pt complex. Temperature‐dependent absorption spectra of the copolymers in dilute dichlorobenzene solution were also recorded to investigate the effect of Pt complexation on the aggregation characteristics of polymers (Figure S4, Supporting Information). As the temperature increased, the intensity of absorption at *λ*_0–0_ gradually weakened, and the 0–1 peak (*λ*_0–1_) became dominant. In addition, the *I*_0–0_/*I*_0–1_ intensity ratio of PM6‐Pt1, PM6‐Pt3, and PM6‐Pt5 decreased more considerably than PM6 with increasing solution temperature, indicating that Pt complexation can lower the molecular aggregation strength (Figure S5, Supporting Information). This controllable aggregation of metalated terpolymers is critical for forming an optimal phase‐separated morphology in OSCs.^[^ ^26^ ^]^ In the solid thin films (Figure 3b), all metalated terpolymers showed nearly the same absorption onsets at ≈685 nm, with similar optical bandgaps (*E*_g_^opt^) of 1.81 eV, and their solid state colors are dark brown. Besides, PM6‐Pt3 exhibited redshifted absorption and a larger *I*_0–0_/*I*_0–1_ ratio compared to other polymer films, indicating stronger aggregation behavior of PM6‐Pt3. This is likely due to the proper amount of Ptpy monomer, in which the molecular interaction dominates the molecular aggregation. However, for PM6‐Pt5, with a higher ratio of Ptpy, the reduced rigidity may weaken the molecular aggregation. The films showed strong absorptions in the range of 400–700 nm, complementing the absorption of the acceptor L8‐BO. Differential scanning calorimetry (DSC) profiles revealed that all polymers exhibited no clear melting and crystallization transitions during heating and cooling scans (in the temperature range of 50—310 °C) (Figure 3c), indicating an amorphous morphology. Cyclic voltammetry (CV) was performed to investigate the electrochemical properties of the polymers in the films (Figure S6, Supporting Information). Compared with PM6 (−5.53 eV), the introduction of the Ptpy block into the PM6 polymer main chain resulted in deeper HOMO energy levels: −5.60, −5.68, and −5.69 eV for PM6‐Pt1, PM6‐Pt3, and PM6‐Pt5, respectively. These results are consistent with the DFT calculations. The LUMO energy levels were calculated to be −3.69, −3.79, −3.87, and −3.88 eV for PM6, PM6‐Pt1, PM6‐Pt3, and PM6‐Pt5, respectively, according to the *E*_LUMO_ = *E*_g_^opt^ + *E*_HOMO_ (Figure 3d). The low‐lying HOMO energy level is beneficial for decreasing the sub‐bandgap energy loss in OSCs and increasing the resulting *V*_oc_ of the devices.

Considering that the Pt complex monomer should be favorable for affording triplet excitons of long lifetimes, the exciton lifetimes of the polymers were further investigated by time‐resolved photoluminescence (TRPL) measurements. As shown in Figure S7 and Table S2 (Supporting Information), the average lifetime (τ_ave_) values were 0.351, 0.943, 1.022, and 1.094 ns for PM6, PM6‐Pt1, PM6‐Pt3, and PM6‐Pt5, respectively, indicating increased lifetimes for the metalated terpolymers. The prolonged lifetime contributes to increased exciton diffusion length, directly impacting the photocurrent generation.^[^ ^29^, ^30^ ^]^ Specially, the τ_ave_ values were not enhanced largely for PM6‐Pt3 and PM6‐Pt5 compared with PM6‐Pt1, which may be influenced by the molecular structure and rigidity of the polymer backbone. The *L*_D_ of the polymer films, measured using the exciton−exciton annihilation method through TAS (Figure 3e; Figure S8 and Table S3, Supporting Information), was 10.68 nm for the control PM6 film, and enhanced to 14.82, 16.64, and 15.13 nm for PM6‐Pt1, PM6‐Pt3, and PM6‐Pt5 films, respectively. The increased *L*_D_ is attributed to the longer exciton lifetime, which restrains charge recombination and improves the *J*_sc_ of the corresponding devices. Besides, the hole transport properties of the polymers were measured using the space charge limited current (SCLC) method (Figure S9 and Table S4, Supporting Information). Compared with PM6 (hole mobility (*μ*_h_) of 2.24 × 10^−4^ cm^2^ V^−1^ s^−1^), the *μ*_h_ values gradually reduced to 1.68 × 10^−4^, 1.61 × 10^−4^, and 1.58 × 10^−4^ cm^2^ V^−1^ s^−1^ for PM6‐Pt1, PM6‐Pt3, and PM6‐Pt5, respectively, resulting from the decreased crystallinity of the metalated terpolymers (vide infra). Steady‐state photoluminescence (PL) measurements were conducted to further study the properties of singlet excitons in thin films of these polymers (Figure S10, Supporting Information). Interestingly, the metalated terpolymers exhibited much higher PL intensity than PM6, suggesting increased radiative decay of the singlet excitons. Furthermore, the photoluminescence quantum yield (PLQY) of these polymers was investigated by using 600 nm light excitation (Figure S11, Supporting Information). The control PM6 showed a very low PL signal with undetected PLQY. In contrast, the metalated terpolymers had PLQY values of 3%, 2.8%, and 2.5% for PM6‐Pt1, PM6‐Pt3, and PM6‐Pt5, respectively. The enhanced PLQY of metalated terpolymers is likely due to the reduction of vibrational coupling between molecules, caused by the Ptpy monomer with its conjugated structure and absence of alkyl chains compared to the BDD unit. The enhanced PLQY can increase the electroluminescence quantum efficiency (EQE_EL_), thus decreasing the non‐radiative recombination loss of OSCs due to the hybridization between singlet excitons and CT states.^[^ ^31^, ^32^, ^33^ ^]^ To further study the decay of the singlet excitons in these polymers, which can undergo ISC process to produce more triplet excitons owing to the heavy metal, the *k*_ISC_ values were calculated (Table S5, Supporting Information). The *k*_ISC_ of PM6‐Pt5 was enhanced to 4.27 × 10^8^ s^−1^, slightly higher than PM6‐Pt1 (*k*_ISC_ of 3.2 × 10^8^ s^−1^) and PM6‐Pt3 (*k*_ISC_ of 3.74 × 10^8^ s^−1^) (Figure 3f), indicating that higher Ptpy content in the backbone facilitates the ISC process. The trend was consistent with the theoretical results by DFT. Overall, these results confirmed that introducing the Ptpy unit into PM6 main chains not only increases the *L*_D_ and improves PLQY of the polymers, but also facilitates ISC process to produce triplet excitons. The long lifetimes of triplet excitons positively impact the reduction of geminate recombination.

**Figure 4:**
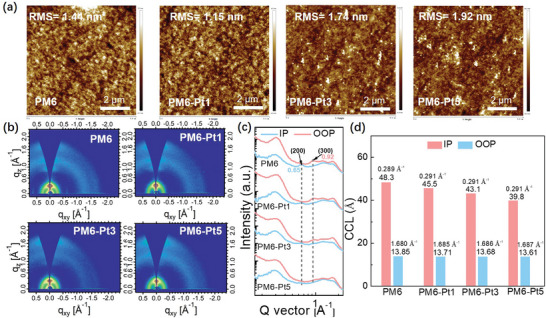
a) Tapping‐mode AFM height images of the polymer films. b) GIWAXS patterns of the pure films. c) Out‐of‐plane and in‐plane line‐cut profiles of the pure films. d) The peaks and corresponding CCL values in the IP and OOP direction of the pure films.

The effect of introducing Pt complex into PM6 on the morphology and molecular packing was further investigated using atomic force microscopy (AFM) and grazing‐incidence wide‐angle X‐ray scattering (GIWAXS). As shown in **Figure** **4**a, the root‐mean‐square (RMS) values of PM6, PM6‐Pt1, PM6‐Pt3, and PM6‐Pt5 films are 1.44, 1.15, 1.74, and 1.47 nm, respectively, indicating a smoother surface for the PM6‐Pt1 film. This smooth surface is attributed to the alleviation of polymer aggregation and the formation of uniform phase. However, as the degree of Pt‐complexation increases further in PM6‐Pt3 and PM6‐Pt5, the rigidity of the polymer chains is impaired and crystallinity is lowered, negatively affecting the morphology. Then, the crystallinity and molecular packing were investigated by GIWAXS. As shown in Figure 4b, all polymers displayed a face‐on orientation, evidenced by the (100) diffractions in the in‐plane (IP) direction and (010) diffractions in the out‐of‐plane (OOP) direction. For the metalated terpolymers, the position of (100) peak slightly increased to 0.291 Å^−1^, corresponding to a reduced lamellar stacking distance of 21.58 Å compared to PM6, which had a (100) peak at 0.289 Å^−1^ with a lamellar stacking distance of 21.73 Å. This reduced lamellar stacking distance is probably due to the strong C─H**^…^** π interaction in adjacent Ptpy molecules. However, the crystalline coherence length (CCL) gradually decreased from 48.3 to 39.8 Å with an increasing degree of Pt‐complexation. What's more, in the PM6 films, the strength of the (200) and (300) diffractions located at q_xy_ = 0.65 and 0.92 Å^−1^ decreased gradually with increasing Ptpy content (Figure 4c). Besides, (010) diffractions slightly shifted from q_z_ = 1.680 to 1.687 Å^−1^, corresponding to a decreased π–π stacking distance from 3.73 to 3.72 Å (Table S6, Supporting Information). The reduced π–π stacking distance of the metalated terpolymers can be ascribed to the strong π–π stacking interaction of the Ptpy monomers observed in the single crystal results. From PM6 to PM6‐Pt5, the CCL of π–π stacking was marginally reduced from 13.85 to 13.61 Å (Figure 4d). These results clearly show that the Pt‐complexation damaged the rigidity of polymer chains, thus lowering the crystallinity and aggregation strength. The Ptpy monomer, with strong π–π stacking and C─H**^…^** π interaction, leads to a reduction in lamellar stacking and π–π stacking distances in the metalated terpolymers. The decreased crystallinity in the polymers with Pt complex is further confirmed by the reduced Raman intensity in the range of 1400–1600 cm^−1^.

### 2.2 Photovoltaic Properties and Energy Loss (*E*_loss_) Analysis

**Figure 5:**
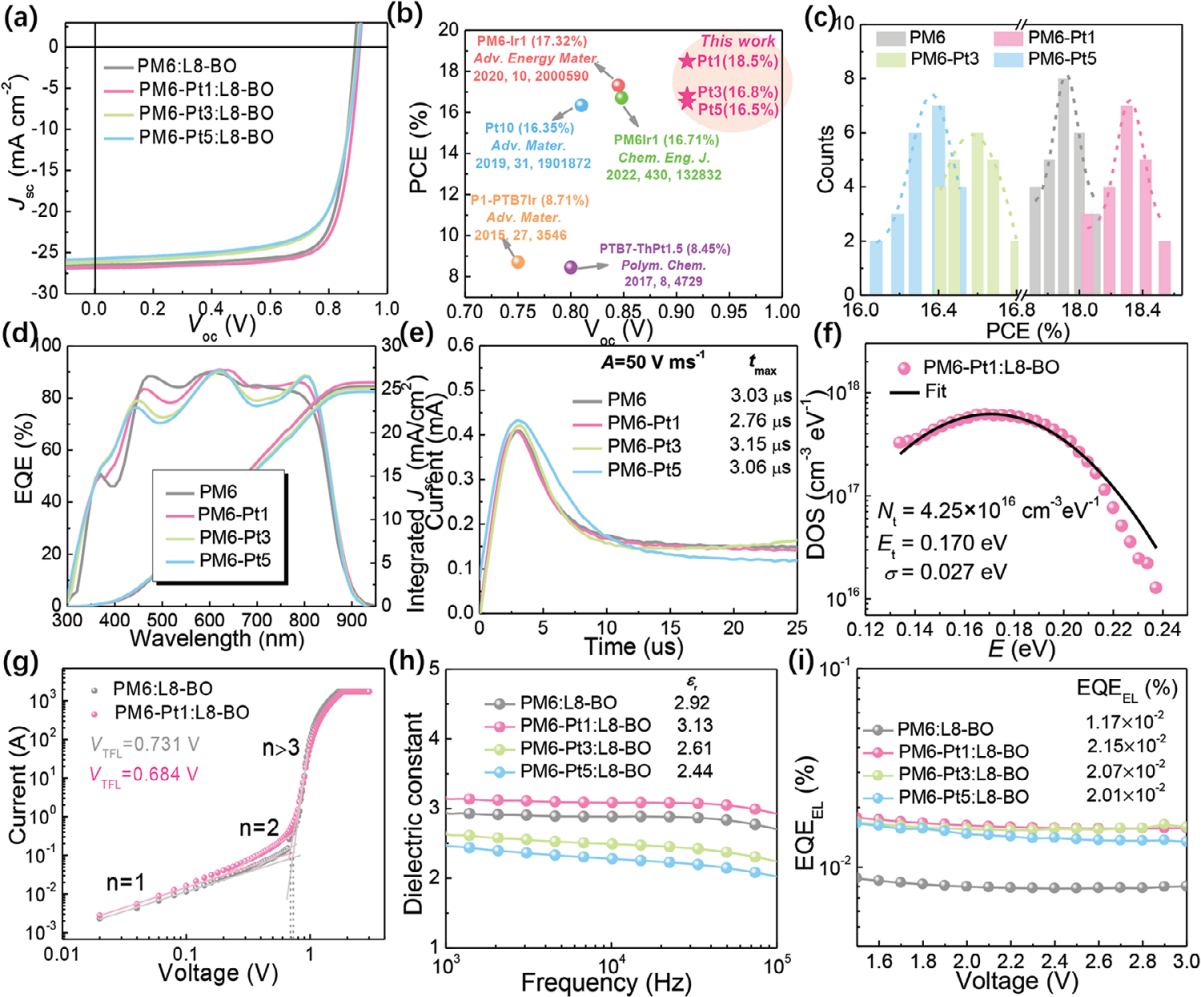
a) *J*−*V* characteristics of the devices based on the polymer donors under AM1.5G illumination (100 mW cm^−2^). b) PCE versus *V*_oc_ of reported metalated terpolymer donors as compared with our work. c) Histogram of the PCEs of the devices. d) EQE spectra of the corresponding devices. e) Photo‐CELIV plots with a voltage ramp of 50 V ms^−1^. f) Trap density of states (tDOS) spectra and corresponding Gaussian fitting (solid line) for PM6‐Pt1:L8‐BO device. g) *J*–*V* characteristics of electron‐only devices based on PM6:L8‐BO and PM6‐Pt1:L8‐BO (*n* represents the slope of the fitting line). h) The relative dielectric constants of the devices at different frequencies. i) The EQE_EL_s of different devices.

The Pt complexation effects on the photovoltaic performance were systematically studied in OSCs using a device structure of ITO/PEDOT:PSS/active layer/PDINN/Ag. The current–density voltage (*J*–*V*) curves and device parameters of the OSCs are presented in **Figure** **5**a and **Table** **2**. The standard PM6:L8‐BO devices exhibited a *V*_oc_ of 0.88 V, a *J*_sc_ of 26.41 mA cm^−2,^ and an FF of 77.88%, delivering a PCE of 18.10%. Notably, the PM6‐Pt1:L8‐BO‐based devices achieved a higher PCE of 18.54%, owing to the simultaneous enhancement in *V*_oc_ (0.91 V) and *J*_sc_ (26.87 mA cm^−2^). To the best of our knowledge, this is the best record for OSCs using metalated terpolymers as the electron donors (Figure 5b). A certified efficiency of 18.32% was achieved by the South China National Center of Metrology‐Guang Dong Institute of Metrology (Figure S13, Supporting Information). However, for the PM6‐Pt3 and PM6‐Pt5‐based devices, despite the increased *V*_oc_ of 0.90 V, inferior *J*_sc_ and FF parameters resulted in PCE of 16.80% and 16.51%, respectively. The increased *V*_oc_ of these devices can be attributed to the lower energy loss and the progressively deepened HOMO levels of these metalated terpolymers compared to PM6. The increased *J*_sc_ in PM6‐Pt1:L8‐BO‐based devices primarily originates from the improved charge transport and lower trap density. As has been discussed above, the molecular weight of the metalated terpolymers was close to each other. In this case, there should be other explanations for the difference in device performance (vide infra). The efficiency distribution histogram of over 20 independent binary devices is shown in Figure 5c. The external quantum efficiency (EQE) spectra of the binary devices are shown in Figure 5d. Compared with PM6:L8‐BO device, PM6‐Pt1:L8‐BO device exhibited lower photo‐responses within 440–600 nm owing to the weaker absorption in this region (Figure S14, Supporting Information). However, in the 620–900 nm range, the PM6‐Pt1:L8‐BO device exhibited a higher EQE response than the PM6:L8‐BO device, indicating higher charge extraction and resulting in an enhanced *J*_sc_. For PM6‐Pt3‐ and PM6‐Pt5‐based devices, the photo‐responses within 440–770 nm were lower than that of the control device, leading to reduced *J*_sc_ in the corresponding OSCs. The integrated *J*_sc_ values of the binary devices are 25.35, 25.79, 25.09, and 24.72 mA cm^−2^ for PM6, PM6‐Pt1, PM6‐Pt3, and PM6‐Pt5 devices, respectively. To further explore the potential of the metalated terpolymers, ternary OSCs were fabricated (Figure S15 and Table S7, Supporting Information). The optimal ternary devices based on PM6‐Pt1:PM6:L8‐BO (0.8:0.2:1.2, wt/wt/wt) achieved a higher PCE of 19.24%, owing to the simultaneous enhancement in *J*_sc_ (27.56 mA cm^−2^) and FF (77.60%) (Table 2).

**Table 2:** Photovoltaic parameters of devices based on PM6, PM6‐Pt1, PM6‐Pt3 and PM6‐Pt5 polymers.

Active layer	*V*_oc_ (V)	*J*_sc_ (mA cm^−2^)	*J*_sc_ (mA cm^−2^)^a)^	FF (%)	PCE (%)^b)^
PM6:L8‐BO	0.88 (0.87 ± 0.01)	26.41 (26.3 ± 0.11)	25.35	77.88 (77.5 ± 0.38)	18.10 (18.00 ± 0.10)
PM6‐Pt1:L8‐BO	0.91 (0.90 ± 0.01)	26.87 (26.41 ± 0.47)	25.79	75.83 (75.1 ± 0.73)	18.54 (18.35 ± 0.19)
PM6‐Pt3:L8‐BO	0.90 (0.89 ± 0.01)	26.14 (26.00 ± 0.14)	25.09	71.41 (71.0 ± 0.41)	16.80 (16.20 ± 0.60)
PM6‐Pt5:L8‐BO	0.90 (0.89 ± 0.01)	25.76 (25.21 ± 0.55)	24.72	71.21 (70.32 ± 0.8)	16.51 (16.01 ± 0.50)
PM6‐Pt1:PM6:L8‐BO^c)^	0.90 (0.89 ± 0.01)	27.56 (27.12 ± 0.44)	∕	77.60 (77.20 ± 0.4)	19.24 (19.02 ± 0.22)
PM6‐Pt1:L8‐BO^d)^	0.91	26.63		75.6	18.32

^a)^ Calculated current densities from EQE curves;

^b)^ Average PCEs from ten devices;

^c)^ The weight ratio was PM6‐Pt1:PM6:L8‐BO = 0.8:0.2:0.2 (wt/wt/wt);

^d)^ Certified in South China National Center of Metrology‐Guang Dong Institute of Metrology (SCM).

To understand the effect of Pt‐complexation on the charge transport in these devices, photo‐CELIV was employed to quantify charge carrier mobility (*µ*) using the formula: *µ* = 2*d* ^2^/[3*At*_max_^2^(1 + 0.36Δ*j*/*j*(0))], where *d* is the active layer thickness, *A* is the voltage ramp, *t*_max_ is the maximum current time, Δ*j* is the peak transient current, and *j*(0) is the displacement current.^[^ ^34^ ^]^ The representative plots and calculated parameters are shown in Figure 5e and Table S8 (Supporting Information). The *µ* values of PM6‐Pt1:L8‐BO was 1.40 × 10^−4^ cm^2^ V^−1^ s^−1^, higher than those of PM6:L8‐BO (1.17 × 10^−4^ cm^2^ V^−1^ s^−1^), PM6‐Pt3:L8‐BO (1.06 × 10^−4^ cm^2^ V^−1^ s^−1^), and PM6‐Pt5:L8‐BO (9.79 × 10^−5^ cm^2^ V^−1^ s^−1^), indicating better charge transport in PM6‐Pt1‐based device. The hole and electron mobilities of the devices were further studied using the SCLC method (Figure S16 and Table S9, Supporting Information). PM6:L8‐BO device exhibited a high *µ*_h_ of 6.7 × 10^−4^ cm^2^ V^−1^ s^−1^ but a relatively low *µ*_e_ of 3.48 × 10^−4^ cm^2^ V^−1^ s^−1^. The unbalanced hole and electron transport can be attributed to the excessive aggregation of PM6, which isolates the L8‐BO domains, leading to poor electron transport.^[^ ^35^ ^]^ The *µ*_h_ values gradually decreased to 5.66 × 10^−4^ cm^2^ V^−1^ s^−1^ for PM6‐Pt1:L8‐BO, 4.43 × 10^−4^ cm^2^ V^−1^ s^−1^ for PM6‐Pt3:L8‐BO, and 3.72 × 10^−4^ cm^2^ V^−1^ s^−1^ for PM6‐Pt5:L8‐BO, due to the lower crystallinity induced by the decreased rigidity of the polymer chains. However, the *µ*_e_ was significantly improved to 5.78 × 10^−4^ cm^2^ V^−1^ s^−1^ for PM6‐Pt1:L8‐BO, 6.34 × 10^−4^ cm^2^ V^−1^ s^−1^ for PM6‐Pt3:L8‐BO, and 6.96 × 10^−4^ cm^2^ V^−1^ s^−1^ for PM6‐Pt5:L8‐BO. The enhanced electron transport likely originates from the optimized morphology with the increased connection of acceptor domains. The enhanced *µ*_e_ of PM6‐Pt1:L8‐BO blend improves the charge transport, contributing to the higher *J*_sc_ in the devices. Impedance spectra and capacitance–voltage (*C–V*) measurements (Figure S17, Supporting Information) were conducted to calculate the trap density of states (tDOS), providing further insight into the charge transport capacity. The related Gaussian shape distribution of trap DOS can be described in the following expression: *N*_t_(*E*) = *N*_t_exp[−(*E*_t_ −*E*)^2^/2*σ* ^2^]/[(2*π*)^1/2^ *σ*], where *N*_t_ is the total trap density, *E*_t_ is the energy center of trap DOS, and *σ* represents the disorder parameter.^[^ ^36^ ^]^ As shown in Figure 5f and Figure S18 (Supporting Information), the PM6‐Pt1:L8‐BO device exhibited the lowest trap DOS of 4.25 × 10^16^ cm^−3^ eV^−1^, indicating reduced charge recombination and a more ordered molecular energy distribution. Additionally, PM6‐Pt1:L8‐BO device had the narrowest distribution with the smallest *σ* value (*σ* = 27 meV), which can suppress deep trap states and facilitate charge transfer.^[^ ^37^, ^38^ ^]^ We also tested the trap density of the PM6:L8‐BO and PM6‐Pt1:L8‐BO devices using the formula: *V*_TFL_ = (*qN*_t_ *L* ^2^)/(2*ε*_0_ *ε*), where *V*_TFL_ is the trap‐filled limit voltage, *q* is the elementary charge, *N*_t_ is the total trap density, *L* is the film thickness, *ε*_0_ and *ε* are the permittivity of free space and relative permittivity of the material, respectively.^[^ ^39^ ^]^ As shown in Figure 5g, PM6‐Pt1:L8‐BO device exhibited a lower *N*_t_ of 2.27 × 10^16^ cm^−3^ compared to PM6:L8‐BO device (*N*_t_ of 2.42 × 10^16^ cm^−3^), suggesting the suppressed charge recombination in the former device. Based on the *C–V* results, the dielectric constants (*ε*_r_) were also investigated according to the equation: *C* = *𝜀*_0_ *𝜀*_r_ *A*∕*d*, where *ɛ*_0_, *A*, and *d* represent the vacuum permittivity (8.85 × 10^−12^ F m^−1^), device area, and thickness of blend film, respectively.^[^ ^40^ ^]^ Then, the binding energies of excitons can be calculated as follows: *E*_B_ = *q* ^2^∕4𝜋r*𝜀*_0_ *𝜀*_r_, where *q* represents the elementary charge, and *r* represents the distance between electron and hole.^[^ ^41^ ^]^ As shown in Figure 5h, the PM6‐Pt1:L8‐BO device has the highest *ε*_r_ of 3.13 with the smallest *E*_B_ of 0.30 eV, thus facilitating exciton dissociation and charge separation.^[^ ^42^ ^]^

To gain insight into the role of the Pt complex on the charge collection (*P*_c_) process in these devices, the dependence of photocurrent density (*J*_ph_) on effective voltage (*V*_eff_) was investigated and described as *J*_ph_/*J*_sat_, where *J*_sat_ is the saturated *J*_ph_.^[^ ^43^ ^]^ The PM6‐Pt1:L8‐BO device achieved the highest *P*_c_ value of 0.971, compared to the other devices (*P*_c_ values of of 0.965, 0.940, and 0.928 for PM6, PM6‐Pt3, and PM6‐Pt5 devices, respectively), demonstrating improved charge dissociation and charge collection at the D/A interface (Figure S19a, Supporting Information). The charge recombination kinetics of the devices were further investigated through the relationship between *J*_sc_ and incident light intensity (*P*_in_), described as *J*_sc_∝*P*_in_^α^.^[^ ^44^, ^45^ ^]^ As shown in Figure S19b (Supporting Information), the α value increased from 0.959 to 0.980 with the inclusion of 1% Ptpy monomer into the polymer, indicating suppressed bimolecular recombination. Similarly, the factor *n* fitted from the dependence of *V*_oc_ on *P*_in_, described as *V*_oc_∝*nk*_B_ *T*/*q* ln(*P*_in_), was evaluated to study trap‐assisted recombination.^[^ ^46^ ^]^ As shown in Figure S19c (Supporting Information), the *n* value was reduced to 1.07 for the PM6‐Pt1:L8‐BO device, indicating suppressed trap‐assisted recombination. We also evaluated the charge extraction capability and charge carrier lifetimes of the devices by testing transient photocurrent (TPC) and transient photovoltage (TPV) measurements, respectively.^[^ ^47^, ^48^ ^]^ As shown in Figure S20a (Supporting Information), the PM6‐Pt1:L8‐BO device exhibited the shortest charge sweepout time of 0.248 µs, suggesting faster charge extraction property. In addition, a longer carrier lifetime was observed in the PM6‐Pt1:L8‐BO device (6.01 µs) compared to the PM6:L8‐BO device (4.47 µs), implying less charge recombination (Figure S20b, Supporting Information). Besides, the PM6‐Pt1:L8‐BO film exhibited the highest PL quenching efficiency of 98.2% among the four systems, indicating efficient charge transfer in the PM6‐Pt1:L8‐BO device (Figure S21, Supporting Information). All these results confirm that PM6‐Pt1 polymer with moderate Pypy content can suppress the charge recombination, improve the charge transfer, and thus support the increased *J*_sc_ in the device.

The *E*_loss_ analysis was conducted to investigate the improved *V*_oc_ of the metalated terpolymers‐based devices compared to the control device. *E*_loss_ can be divided into three contributions: 1) radiative recombination from the absorption above the bandgap (∆*E*_1_ = *E*_g_ − *qV*_oc_^SQ^), 2) additional radiative recombination from the absorption below the bandgap (∆*E*_2_ = *qV*_oc_^SQ^ – *qV*_oc_^rad^), and 3) non‐radiative recombination loss (Δ*E*_3_ = − *kT*In(EQE_EL_)).^[^ ^27^ ^]^ As reported in the literature, the main *E*_loss_ of OSCs is derived from Δ*E*_3_, which is directly determined by the EQE_EL_.^[^ ^27^ ^]^ Here, we mainly focused on studying Δ*E*_3_ to explore the reasons for increased *V*_oc_ in the metalated terpolymers‐based devices. As plotted in Figure 5i, higher EQE_EL_ values of 2.15 × 10^−4^, 2.07 × 10^−4^, and 2.01 × 10^−4^ are observed for PM6‐Pt1, PM6‐Pt3, and PM6‐Pt5‐based devices, respectively, which are higher than that of PM6‐based device (EQE_EL_ of 1.17 × 10^−4^). These results are consistent with the PLQY of the polymers discussed earlier, indicating that enhancing the PLQY of component is a feasible method to increase EQE_EL_. As a result, the PM6‐based devices suffered from a relatively high ∆*E*_3_ of 0.234 eV. In contrast, ∆*E*_3_ was significantly lowered to 0.219, 0.220, and 0.220 eV for PM6‐Pt1, PM6‐Pt3, and PM6‐Pt5 devices, respectively. This result indicates that the introduction of Pt complex can reduce the non‐radiative recombination energy loss, thus offering higher *V*_oc_ for the corresponding device.

**Figure 6:**
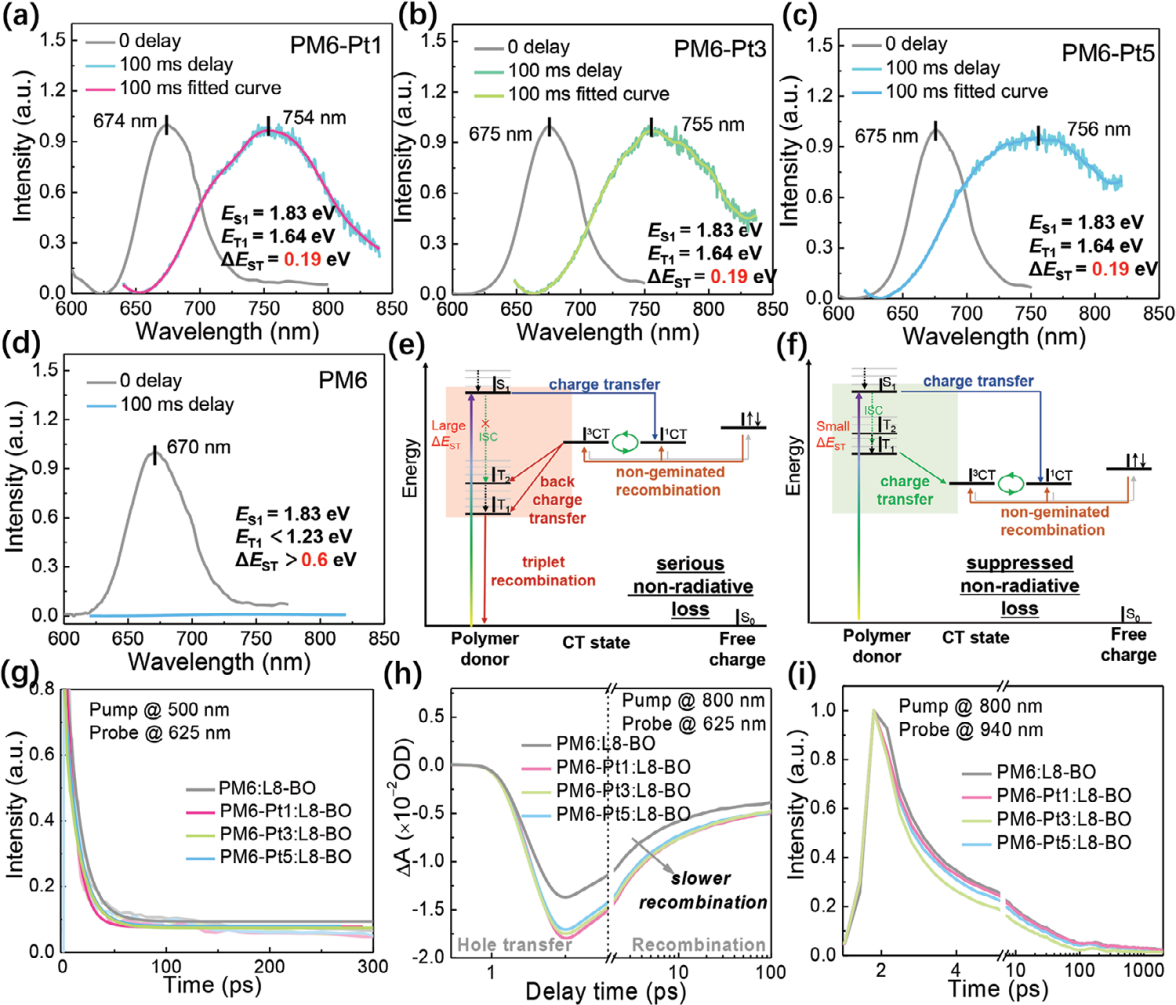
PL emission spectra of the polymer films at 77 K with different delay times: a) PM6‐Pt1, b) PM6‐Pt3, c) PM6‐Pt5, and d) PM6. Jablonski diagram of the electronic states in the polymers: e) T_1_ state below the CT state and f) T_1_ state above the CT state. g) The decay traces of GSB at 625 nm for the blend films with an excitation wavelength of 500 nm. h) The decay traces of GSB at 625 nm for the blend films with an excitation wavelength of 800 nm. i) The decay traces of ESA at 940 nm for the blend films with an excitation wavelength of 800 nm.

To further study the non‐radiative recombination energy loss in these devices, the triplet states were investigated by the fluorescent and phosphorescent emission at 77 K. As shown in **Figure** **6**a–c, the metalated terpolymers showed fluorescent and phosphorescent emission peaks at around 674 and 755 nm, corresponding to *E*_S1_ and *E*_T1_ of 1.83 and 1.64 eV, respectively. Therefore, the ∆*E*_ST_ values of the metalated terpolymers were estimated to be 0.19 eV. Obviously, the small ∆*E*_ST_ value is favorable for the ISC process of excitons from S_1_ to T_1_. In contrast, for PM6, only a fluorescent emission peak was observed at 670 nm with *E*_S1_ of 1.83 eV. The phosphorescent emission of PM6 was hardly detected due to its large ∆*E*_ST_ (> 0.6 eV), which energetically blocks the ISC process from S_1_ to T_1_ (Figure 6d).^[^ ^20^ ^]^ These results are consistent with the *k*_ISC_ analysis, where the metalated terpolymers exhibited considerable *k*_ISC_ values, while the *k*_ISC_ of PM6 polymer was negligible owing to its low PLQY. In the analysis of *E*_loss_, the energy levels of the CT state (*E*_CT_) in these devices were estimated to be 1.36, 1.38, 1.38, and 1.37 eV for PM6, PM6‐Pt1, PM6‐Pt3, and PM6‐Pt5‐based devices, respectively, from the sEQE and EL results (Figure S22, Supporting Information). This suggests that the back transfer from the CT state to the triplet state is allowable for PM6, inducing serious non‐radiative recombination loss via triplet recombination in the devices (Figure 6e). In contrast, the *E*_T1_ values of the metalated terpolymers are higher than the corresponding *E*_CT_, thus preventing the non‐radiative recombination via triplet states (Figure 6f). These results indicate that Pt‐complexation not only elevates *E*_T1_ to promote ISC for generating more triplet excitons with longer lifetimes, which reduces geminate recombination and benefits charge transport in these metalated terpolymers devices but also effectively prohibits back transfer from the CT state to the triplet state, thereby restricting non‐radiative recombination loss.

Charge transfer and recombination kinetics were further clarified using the transient absorption spectroscopy (TAS) measurement.^[^ ^29^, ^30^, ^49^ ^]^ A 500 nm pump beam was used to excite the blend films to study the electron transfer process (Figure S23, Supporting Information). The GSB signals of the polymers and L8‐BO were individually monitored in the range of 600–650 nm and 780–820 nm, respectively. The kinetics at 625 nm in the blends were extracted and fitted with a double exponential decay function (Figure 6g). The PM6‐Pt1:L8‐BO film exhibited faster electron transfer with the shortest carrier lifetime of 10.02 ps (Table S10, Supporting Information). Moreover, both shorter τ_1_ and τ_2_ values were obtained for PM6‐Pt1:L8‐BO device, implying more rapid exciton dissociation at the donor–acceptor interface and faster exciton diffusion in the ternary devices.^[^ ^50^ ^]^ Then, the hole transfer dynamics were studied using an 800 nm wavelength to excite the blend films. All donor GSB signals at 625 nm gradually increased in the blend films, indicating efficient hole transfer from the acceptor to the donors (Figure S24, Supporting Information).^[^ ^51^ ^]^ The kinetics at 625 nm of the blend films were extracted (Figure 6h). The PM6‐Pt1:L8‐BO blend exhibited faster hole transfer and slower recombination compared to PM6:L8‐BO. What's more, the signals at ≈940 nm represented the photoinduced absorption (PIA) of L8‐BO singlet excitons. PM6‐Pt1:L8‐BO exhibited a faster singlet exciton decay signal than PM6‐L8‐BO (Figure 6i). The shorter lifetime of the PM6‐Pt1‐based film shows that the singlet excitons in L8‐BO undergo hole transfer more rapidly to the donor PM6‐Pt1, facilitating effective exciton dissociation, which agrees well with the TA results. The TA experiments further explain the higher *J*_sc_ of PM6‐Pt1:L8‐BO devices. As shown in Figure S25 (Supporting Information), PM6‐Pt1:L8‐BO shows the shortest τ_ave_ value of 0.249 ns in the TRPL results, indicating the fastest charge transfer and accounting for the high *J*_sc_ (Table S11, Supporting Information).

### 2.3 Morphology Properties

**Figure 7:**
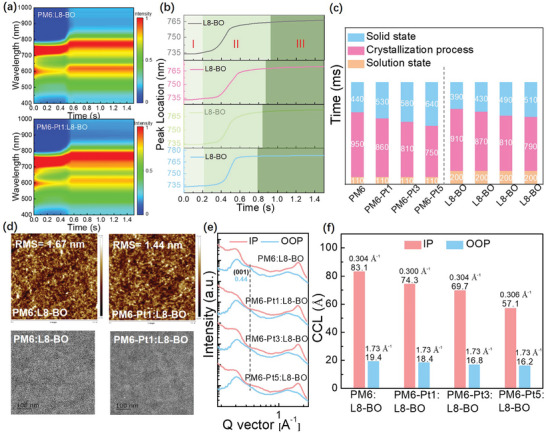
a) Time‐resolved UV–vis absorption spectra of PM6:L8‐BO and PM6‐Pt1:L8‐BO blend films. b) The peak position evolution as a function of time for the blend films. Decay dynamics of excitons of L8‐BO. c) The abridged graph of four blend films during the film‐forming process. d) Tapping‐mode AFM height images and TEM images of the corresponding binary blend films. e) Out‐of‐plane and in‐plane line‐cut profiles of the blend films. f) The peaks and corresponding CCL values in the IP and OOP direction of the blend films.

The film formation process of the polymer donors and L8‐BO was studied using in‐situ UV–vis absorption spectra.^[^ ^52^, ^53^ ^]^ As shown in **Figures** **7**a and S26 (Supporting Information), PM6 exhibits a crystallization time (stage II) of 0.95 s in the PM6:L8‐BO (Figure 7c; Figure S27, Supporting Information). In contrast, the metalated terpolymers undergo gradually decreased crystallization time of 0.86 s, 0.81 s, and 0.75 s for PM6‐Pt1, PM6‐Pt3, and PM6‐Pt5, respectively. These results suggest that Pt‐complexation disturbs the rigidity of polymer chains, delaying the self‐aggregation, shortening the stacking time and reducing the crystallinity. The results are consistent with the GIWAXS analysis of pure polymer films. Besides, the crystallization time of the L8‐BO acceptor in these devices is slightly reduced (Figure 7b), indicating that mitigated coplanarity of the Pt‐based polymers by enhancing the degree of Pt complexation slightly reduces the crystallinity and aggregation strength of L8‐BO molecule in the corresponding blends.^[^ ^26^ ^]^ To further investigate the morphology, the miscibility between the polymer donor and L8‐BO was evaluated using the surface energy analysis (Figure S28 and Table S12, Supporting Information). The interaction parameter χ, calculated as χ = *k*($\sqrt{\gamma_{A}}$−$\sqrt{\gamma_{B}}$)^2^, where *k* is a positive constant, and *γ*_A_ and *γ*_B_ refer to the surface tension of A and B in their neat films, was used to assess the miscibility.^[^ ^54^ ^]^ Compared to the pure PM6 film, the surface energy of metalated terpolymers increases slightly. Specially, the χ value is found to be χ_PM6‐Pt1/L8‐BO_ = 0.014 K, which is smaller than χ_PM6/L8‐BO_ = 0.033 K, suggesting that PM6‐Pt1 exhibits enhanced compatibility with L8‐BO, promoting the formation of a well‐mixed phase in the blends. Then, the morphology was further investigated using atomic force microscopy (AFM), transmission electron microscopy (TEM) and GIWAXS characterization. As shown in Figure 7d, PM6:L8‐BO and PM6‐Pt1:L8‐BO exhibit smooth surfaces with root‐mean‐square (RMS) roughness values of 1.67 and 1.44 nm, respectively. The reduced roughness of the latter film may result from the suppressed aggregation of polymer and enhanced miscibility between PM6‐Pt1 and L8‐BO. However, the enhanced degree of Pt complexation in the PM6‐Pt3 and PM6‐Pt5 blend films significantly increases the roughness, indicating that excessive Pt complexation can seriously damage the morphology (Figure S29, Supporting Information). TEM images (Figure 7d) demonstrated that PM6‐Pt1:L8‐BO film exhibited sparse dark aggregate regions compared to PM6:L8‐BO, implying reduced polymer aggregation. Nevertheless, both PM6:L8‐BO and PM6‐Pt1:L8‐BO films exhibited well‐defined phase separation and nanoscale domains suitable for effective charge generation and carrier transport properties, as demonstrated by their high FFs (over 75%) and EQEs (over 80%) in the devices. Then, the crystallinity of the blend films was further studied through GIWAXS analysis.^[^ ^55^, ^56^ ^]^ All the blend films exhibited face‐on orientation for efficient vertical charge transfer (Figure S30, Supporting Information). The PM6:L8‐BO film showed a large CCL of 83.1 Å in the in‐plane (IP) direction. However, the CCL (IP direction) of the metalated terpolymers‐based films was decreased from 74.36 Å for PM6‐Pt1:L8‐BO to 57.09 Å for PM6‐Pt5:L8‐BO film (Figure 7f). A similar trend was observed in the out‐of‐plane (OOP) direction (Table S13, Supporting Information). Notably, an intense (001) peak at 0.44 Å^−1^ in the q_xy_ direction was discerned for the PM6:L8‐BO, attributed to L8‐BO acceptor (Figure 7e). This peak gradually disappeared in the metalated terpolymer blend films, indicating that the molecular stacking order of L8‐BO and its crystallinity were slightly reduced by introducing Pt‐incorporated polymers. Obviously, these results suggest that the alleviated rigidity of the Pt‐based polymers decreases the crystallinity and self‐aggregation of polymer donors and L8‐BO in the blends. All these results are consistent with the above film formation analysis, and further confirmed by the Raman intensity shown in Figure S31 (Supporting Information). Raman bands at ≈2225 cm^−1^, assigned to the *ν*(C≡N) vibration from L8‐BO, were significantly decreased in intensity in the metalated terpolymers‐based films, indicating lower crystallinity of the acceptors.

To explore the universality of PM6‐Pt1 polymer, we examined its general applicability in other systems. Small molecular acceptors Y6, eC9, and polymer acceptor PY‐DT were chosen to construct the OSCs. The optimal device parameters are summarized in **Table** **3**, and the corresponding *J*–*V* curves are shown in Figure S32 (Supporting Information). The best PCEs achieved were 18.02%, 18.75% and 17.27% for PM6‐Pt1:Y6, PM6‐Pt1:eC9, and PM6‐Pt1:PY‐DT devices, respectively. Obviously, all PM6‐Pt1‐based devices exhibited improved PCE with the significantly increased *V*_oc_. The enhanced *V*_oc_ of the devices can be attributed to the reduced non‐radiative recombination. Additionally, improved charge transport and lower trap density contributed to the increased *J*_sc_ and FF in the PM6‐Pt1‐based devices. These results demonstrate the general applicability of PM6‐Pt1, providing an effective method to construct high‐performance and low energy loss devices by incorporating a Pt complex into the polymer chains.

**Table 3:** Photovoltaic parameters of devices based on PM6 and PM6‐Pt1 polymer donors and different acceptors.

Active layer	*V*_oc_ (V)	*J*_sc_ (mA cm^−2^)	FF (%)	PCE (%)^a)^
PM6:Y6	0.861	26.90	74.18	17.18
PM6‐Pt1:Y6	0.880	27.45	74.60	18.02
PM6:eC9	0.850	27.68	75.86	17.75
PM6‐Pt1:eC9	0.880	27.89	76.41	18.75
PM6:PY‐DT	0.980	23.15	72.50	16.44
PM6‐Pt1:PY‐DT	0.990	23.86	73.13	17.27

^a)^ Average PCEs from five devices.

The impact of the Pt complex on the device stability was further investigated at the maximum power point (MPP) under white light in a N_2_ glovebox. As displayed in Figure S33a (Supporting Information), the PM6:L8‐BO device experienced a rapid burn‐in loss, with its PCE dropping to 80% of the initial value within 334 h (*T*_80_). In contrast, the PM6‐Pt1:L8‐BO device exhibited a slower burn‐in loss, maintaining 84.8% of its initial PCE after 700 h at MPP, showcasing its potential for long‐term photostability. Furthermore, we also investigated the thermal stability of the devices by continuous heating at 70 °C in a N_2_ atmosphere. To avoid the adverse effects of interface layer degradation, inverted devices with the structure of ITO/ZnO/Active layer/MoO_3_/Ag were constructed. As shown in Figure S33b (Supporting Information), the extrapolated *T*_80_ lifetime of PM6‐Pt1:L8‐BO device was up to 1270 h, while the PM6:L8‐BO device exhibited *T*_80_ of only 305 h, suggesting a significant enhancement in long‐term thermal stability of the former devices. These results indicate that the metalated terpolymer PM6‐Pt1, with its slightly reduced polymer chain rigidity and self‐aggregation, significantly contributes to the long‐term photostability and thermal stability of the devices.

## 3 Conclusion

In conclusion, we have reported the design and synthesis of a set of terpolymers by introducing a simple Pt complex block into the PM6 polymer backbone. Owing to the heavy atom effect, these targeted terpolymers exhibited high *E*_T1_ (≈1.64 eV) and small ∆*E*_ST_ (≈0.19 eV), facilitating the ISC process for the generation of triplet excitons. As a result, the metalated terpolymers showed prolonged exciton lifetime and diffusion length for better charge transport. Non‐radiative recombination via terminal triplet loss channels was effectively suppressed. Additionally, the introduction of Pt complex modulated polymer self‐aggregation, which optimizes the morphology of the active layer. An impressive PCE of 18.54% was afforded by PM6‐Pt1 in OSCs, representing the highest efficiency for metalated terpolymers to date. Remarkably, a record high PCE of 19.24% was achieved in the PM6‐Pt1:PM6:L8‐BO (0.8:0.2:1.2, wt/wt/wt) ternary devices. Our results demonstrate that designing terpolymers with high *E*_T1_ by introducing a simple metal complex can regulate molecular aggregation and reduce the non‐radiative losses toward achieving the high performance of OSCs.

## Conflict of Interest

The authors declare no conflict of interest.

## Supporting information



Supporting Information

Supporting Information

## Data Availability

The data that support the findings of this study are available from the corresponding author upon reasonable request.
