# Supplementary material for: Small Singlet–Triplet Gap Terpolymer Donor with a Simple Pt Complex Enables Organic Solar Cells with Low Energy Loss and Over 19.2% Efficiency
Source: Adv Sci (Weinh). 2025 Feb 6;12(12):2410154. doi: 10.1002/advs.202410154 (PMC11948028; doi:10.1002/advs.202410154)
Supplement: Supplementary file 1 — Supporting Information [file ADVS-12-2410154-s001.docx]

Supporting Information

**Small Singlet-Triplet Gap Terpolymer Donor with a Simple Pt Complex Enables Organic Solar Cells with Low Energy Loss and over 19.2% Efficiency**

Dou Luo, Lifu Zhang, Lanqing Li, Tingting Dai, Erjun Zhou, Mao Quan, Hongyang Zhang,* Aung Ko Ko Kyaw,* and Wai-Yeung Wong*

D. Luo, H. Zhang, W.-Y. Wong

Department of Applied Biology and Chemical Technology and Research Institute for Smart Energy

The Hong Kong Polytechnic University

Hung Hom, Hong Kong, P. R. China

E-mail: [wai-yeung.wong@polyu.edu.hk](mailto:wai-yeung.wong@polyu.edu.hk)

A. K. K. Kyaw

Guangdong University Key Laboratory for Advanced Quantum Dot Displays and Lighting Department of Electronic & Electrical Engineering

Southern University of Science and Technology

Shenzhen 518055, P. R. China

E-mail: [aung@sustech.edu.cn](mailto:aung@sustech.edu.cn)

L. Zhang,

Institute of Advanced Scientific Research (iASR)/Key Lab of Fluorine and Silicon for Energy Materials and Chemistry of Ministry of Education

Jiangxi Normal University

Nanchang 330022, P. R. China

L. Li

School of Pharmacy and Food Engineering

Wuyi University

Jiangmen 529020, P. R. China

T. Dai, E. Zhou

National Center for Nanoscience and Technology

Beijing 100190, P. R. China

Mao Quan

College of Materials Science and Engineering, Shenzhen University

Xueyuan Blvd 1066

Shenzhen 518055, P. R. China.

D. Luo, H. Zhang, W.-Y. Wong

The Hong Kong Polytechnic University Shenzhen Research Institute

Shenzhen 518057, P. R. China

**1.1 General Measurements and Characterization**

^1^H NMR and ^13^C NMR spectra were recorded on a Bruker Ascend 400 MHz spectrometer. High-resolution mass spectra were obtained with Thermo Scientific^TM^ Q-Exactive. Elemental analyses (EAs) of compounds were performed on Vario EL cube with CHNS pattern in Fudan University (Shanghai, China).

UV-Vis-NIR absorption spectra were recorded on a Shimadzu UV-3600 UV-Vis-NIR spectrometer. The PL spectra were measured by using a HORIBA LabRAM HR Evolution spectrometer and 532 as an excitation source. The neat PM6, PM6-Pt1, PM6-Pt3 and PM6-Pt5, PM6:L8-BO, PM6-Pt1:L8-BO, PM6-Pt3:L8-BO and PM6-Pt5:L8-BO films were spin-cast on quartz glass from 12 mg mL^−1^ CHCl_3_ solution (total concentration) at a speed of 2000 rpm. Raman spectra were tested using a HORIBA LabRAM HR Evolution spectrometer and 532 nm laser as an excitation source. PLQY spectra were obtained using an absolute PL quantum yield spectrometer (Hmamatsu, Quantaurus-QYC11347-12).

Atomic force microscopy (AFM) measurements were carried out using a NanoMan VS microscope in the tapping mode. TEM images were obtained from a JEM-2100F instrument.

**1.2 Fabrication and Characterization of Organic Solar Cells**

All devices were fabricated based on the conventional structure: ITO/PEDOT:PSS/active layer/PDINN/Ag. ITO-coated glass substrates were cleaned by sonification in acetone, detergent, deionized water, and isopropyl alcohol and dried in a nitrogen stream. The pre-cleaned ITO substrates were coated with PEDOT:PSS (filtered through a 0.45 µm PES filter) by spin-coating (4000 rpm for 30 s, thickness of ∼30 nm) and then baked at 150 °C on a hotplate for 15 min in air. The PEDOT:PSS-coated ITO substrates were transferred into a N_2_-filled glove box for subsequent steps. The total concentration of donor:L8-BO (D:A=1:1.2 weight ratio) was fixed at 16 mg mL^−1^, and the blend films were obtained by spin coating the solutions at 3000 rpm for 30s in chloroform containing 0.5% 1-chloronaphthalene (CN) (v:v 99.5:0.5) as additive. All the optimized thickness is approximately 100 nm as measured by the profilometer. Before spin-coating the electron transporting layer, all the active layers were thermally annealed at 100 °C for 10 min. Finally, 5 nm of the functionalized perylene diimide (PDINN) (1 mg mL^−1^ in methanol) was spin-coated at 3000 rpm for 30 s on the active layer followed by the deposition of 100 nm Ag cathode under a under high vacuum (< 2 ×10^−4^ Pa). All the active device areas were 0.078 cm^2^ through a shadow mask. The current density−voltage (*J*−*V*) curves were measured using Keithley 2400 source meter under 1 Sun (AM 1.5 G spectrum) generated from AAA class solar simulator (Japan, SAN-EI, XES-40S1). The external quantum efficiency (EQE) spectra were measured using a Solar Cell Spectral Response Measurement System QE-R3011 (Enlitech Co., Ltd.). The light intensity at each wavelength was calibrated using a standard single crystal Si photovoltaic cell.

**1.3 Fabrication of Single-Carrier Devices**

Single-carrier device (ITO/PEDOT:PSS(40 nm)/active layer/MoO_3_(10 nm)/Ag and ITO/ZnO(40 nm)/active layer/PDINN/Ag) were fabricated to measure hole and electron mobilities of the blend films, respectively. The active layers were similar to those in the fabrication of OSCs devices. The thickness was approximately 100 nm as measured by the profilometer for all the binary and ternary films. The as-cast pure films of PM6, PM6-Pt1, PM6-Pt3 and PM6-Pt5 were spin-cast from chloroform solution (total concentration, 10 mg mL^−1^) at 2200-2400 rpm for 30 s. The thickness is approximately 60 nm.

The mobility *μ* was derived from the SCLC model which is described by the equation *J* = (9/8)*ε*_0_*ε*_r_*μ*(*V*^2^/*d*^3^),^[1]^ where *J* is the current, *ε*_0_ the permittivity of free space, *ε*_r_ the relative permittivity of the material, *d* the thickness of the active layers, and *V* the effective voltage.

**1.3 Computational Data**

Geometry optimizations were performed with Gaussian 16 A.03 software package. Frequency calculations were then conducted at the same computational level to confirm the nature of all located stationary points and to obtain the thermal correction to Gibbs free energy. The electronic structure and properties of materials in triplet excited state were described by using the spin-unrestricted DFT (UDFT). The spin-orbit coupling (SOC) was calculated by using ORCA 5.0.1 software package. The intersystem crossing rate constant *k*_ISC_ was calculated via FCClasses3 Program.

**1.4** **Analysis of Rate Constants**

The rate constants of radiative decay (*k*_r,s_) and nonradiative decay (*k*_nr,s_) from S1 to S0 states, and the rate constants of intersystem crossing (*k*_ISC_) were calculated from the following equations: *k*_p_ =1/*τ*_p_ (1), *k*_d_ =1/*τ*_d_ (2), *k*_r,s_ = *Ф*_p_*k*_p_ + *Ф*_d_*k*_d_ (3), *k*_nr,s_ = ((1– *Ф*_PL_)/ *Ф*_PL_) *k*_r,s_ (4), *k*_ISC_= *k*_p_ – *k*_r,s_ – *k*_nr,s_ (5), where *τ*_p_ and *τ*_d_ represent the prompt and decay fluorescence lifetimes, which can be determined from the transient PL spectra. The *k*_p_ and *k*_d_ represent the decay rate constants for prompt and delayed fluorescence, respectively. *Ф*_p_ and *Ф*_d_ indicate the prompt and delayed fluorescence components and can be distinguished from the total *Ф*_PL_ by comparing the integrated intensities of the prompt and delayed components in the transient PL spectra.^[2]^

**1.5 Photo-CELIV Test**

Photo-CELIV was conducted on all-in-one platform of Paios (Fluxim AG). A light pulse with a duration of 50 μs generated from a white LED lamp (light intensity 100%) was applied prior to a voltage ramp from 0.05 to 1 V μs^–1^. The charge carrier mobility (μ) was calculated from the equation, *μ* = 2*d*^2^/[3A*t*_max_^2^(1+0.36Δ*j*/*j*(0))], where *d* is the active layer thickness, *A* is the voltage ramp, *t*_max_ is the maximum current time, Δ*j* is the peak transient current, and *j*(0) is the displacement current.

**1.6 Capacitance–Voltage Characterization**

*C*–*V* curve was performed with a frequency analyzer module-equipped potentiostat (PGSTAT302N, Autolab) in dark conditions. A small AC perturbation of 20 mV at a fixed frequency of 10 kHz was applied. The Mott–Schottky curves were obtained by analyzing the data in PIAOS software.

**1.7 Trap Density of States (tDOS)**

Trap density of states (tDOS) were derived from the capacitance−frequency (*C*−*f*) characteristics with *f* ranging from 50 KHz to 5 MHz. The frequency axis (*f*) was transformed to the energy axis (*E*_ω_) from the formula, *E_ω_* = *kTln*(2*ν*_0_/*ω*), where *ν*_0_ is the attempt-to-escape frequency (10^9^ Hz), and *ω* is the angular frequency (*ω* = 2π*f*).^[3]^ The tDOS at energy *E*_ω_, i.e., *N*_t_(*E*_ω_), is associated with the derivative of capacitance (*C*) relative to frequency (*ω*), *N*_t_(*E*_ω_) = – (*V*_bi_/*qL*)*(*dC*/*d*ω)*(ω/*kT*), where *L* is the active layer thickness, and *V*_bi_ is the build-in potential, which can be obtained from Mott-Schottky plot, 1/C^2^ = 2(*V*_bi_-V)/A^2^e*ε*_0_*εN*_A_, where *N*_A_ is the doping parameter, *ε*_0_ is the vacuum permittivity, and *ε* is the relative permittivity. The energy distribution of tDOS was fitted by a Gaussian expression, *N*_t_(*E*) = – $\frac{N_{t}}{\sqrt{2\pi}\sigma}exp[-\frac{{(E_{t}-E)}^{2}}{2\sigma^{2}}]$, where *N*_t_ is the total density, *E*_t_ is the center of the tDOS, and σ is the disorder parameter.

**1.8 Calculation processes of exciton diffusion length**

Exciton diffusion length can be determined by the exciton annihilation method.^[4]^ The pump fluence-dependent TA spectroscopy was used to investigate the diffusion and non-radiative recombination behaviors of excitons in both pure and binary films. It is assumed that trap-induced recombination is the only carrier decay channel under extremely low light fluence: $\frac{dn(t)}{dt}= -kn(t)$ (1), where *n*(*t*) is the charge carrier density as the function of time, and *k* is the rate of trap-induced recombination. The solution of the equation is a single exponential decay function. Then the *k* and the single exponential decay function lifetime τ will follow the equation: *k* = $\frac{1}{}$ (2). Under the extremely low light fluence, the τ of PM6, PM6-Pt1, PM6-Pt3 and PM6-Pt5 are 262.06, 354.21, 442.37 and 317.36 ps. Therefore, the corresponding *k* values are 3.81 × 10^9^, 2.82 × 10^9^, 2.26 × 10^9^ and 3.15 × 10^9^ s^−1^, as shown in Table S2. When the excitation light is sufficiently high to generate enough charge to raise the bimolecular recombination, the charge density decays as follows: $-\frac{dn\left( t \right)}{dt}=kn\left( t \right)+\frac{1}{2}n^{2}(t)$ (3), which has the following solution:$n\left( t \right)=\frac{n(0)e^{-kt}}{1+\frac{}{2k}n\left( 0 \right)[1-e^{-kt}]}$ (4), where *γ* is the singlet-singlet bimolecular exciton annihilation rate. The exciton decays can be well fitted by Eq. (4), where the only free parameter is the bimolecular rate constant. The bimolecular rate constant is then used in the equation: $D= \frac{}{8\pi R}$ (5), in which *D* is the diffusion coefficient, *R* is the annihilation radius of singlet exciton, and here we used the generally assumed value of 1 nm.^[4]^ The exciton diffusion length can be calculated by the following equation:$L_{D}=\sqrt{D}$ (6). (*L*_D_ is the exciton diffusion length, and τ is the lifetime of exciton, which is equal to 1/*k*).

**1.9 Femtosecond (fs) Transient Absorption (TA) Spectroscopy Characterization**

For femtosecond transient absorption spectroscopy, the fundamental output from Yb:KGW laser (1030 nm, 220 fs Gaussian fit, 100 kHz, Light Conversion Ltd) was split into two light beams. One was introduced to NOPA (ORPHEUS-N, Light Conversion Ltd) to produce a certain wavelength for pump beam, the other was focused onto a YAG plate to generate white light continuum as the probe beam. The pump and probe overlapped on the sample at a small angle less than 10°. The transmitted probe light from the sample was collected by a linear CCD array. A pump pulse of 800 nm (below 5 μJ/cm^2^) was employed to excite only acceptors and after a certain delay time, the relative transmittance change (ΔA) was probed using a white-light continuum. The primary absorption peaks for different acceptors and donors are well separated in the spectral domain, therefore, both the spectral and temporal characteristics of hole transfer dynamics can be extracted.

**1.10 Contact Angle Measurements**

The contact angle tests were performed on a Dataphysics OCA40 Micro surface contact angle analyzer. The surface energy of the polymers was characterized and calculated by the contact angles of the two probe liquids with the Wu model.^[5]^

$\frac{\text{4}\text{γ}_{\text{water}}^{\text{d}}\text{γ}_{\text{s}}^{\text{d}}}{\text{γ}_{\text{water}}^{\text{d}}\text{ +}\text{ γ}_{\text{s}}^{\text{d}}}$ + $\frac{\text{4}\text{γ}_{\text{water}}^{\text{p}}\text{γ}_{\text{s}}^{\text{p}}}{\text{γ}_{\text{water}}^{\text{p}}\text{ +}{\text{ }\text{γ}}_{\text{s}}^{\text{p}}}$ = $\text{γ}_{\text{water}}$(1+cos$\text{θ}_{\text{water}}$)

$\frac{\text{4}\text{γ}_{\text{oil}}^{\text{d}}\text{γ}_{\text{s}}^{\text{d}}}{\text{γ}_{\text{oil}}^{\text{d}}\text{ +}\text{ γ}_{\text{s}}^{\text{d}}}$ + $\frac{\text{4}\text{γ}_{\text{oil}}^{\text{p}}\text{γ}_{\text{s}}^{\text{p}}}{\text{γ}_{\text{oil}}^{\text{p}}\text{ +}{\text{ }\text{γ}}_{\text{s}}^{\text{p}}}$ = $\text{γ}_{\text{oil}}$(1+cos$\text{θ}_{\text{oil}}$)

where $\text{γ}_{\text{s}}$ is the total surface energy of acceptors and polymers, and $\text{γ}_{\text{s}}^{\text{d}}$ and $\text{γ}_{\text{s}}^{\text{p}}$ are the dispersion and polar components of $\text{γ}_{\text{s}}$, the values of $\text{γ}_{\text{water}}^{\text{d}}$, $\text{γ}_{\text{water}}^{\text{p}}$, $\text{γ}_{\text{oil}}^{\text{d}}$, $\text{γ}_{\text{oil}}^{\text{p}}$ could be found from the literature and *θ* is the droplet contact angle between sample and probe liquid.

**1.11 In-situ UV-Vis Absorption Spectrum**

In-situ UV-vis absorption spectrum was performed by the Filmetrics F20-EXR spectrometer using the transmission mode with a resolution of 0.1 s. The spectrometer consists of a light source and a detector. The light source and detector are fixed above and below the substrate, respectively, and on the same vertical line. The detector collects the transmission spectra ranging from 400 to 1050 nm during coating. The UV-vis absorption spectra are calculated from the transmission spectra according to the equation A_λ_ = −log_10_(T), where A_λ_ is the absorbance at a certain wavelength (λ) and T is the calculated transmittance. The light source and detector were turned on before coating the film, so time zero is the point when the first solution transmission spectrum was collected by the detector. Before time zero, there is only noise in the transmission spectra.

**1.12 Highly Sensitive EQE and EQE_EL_ Measurements**

Highly sensitive EQE was measured using an integrated system (PECT-600, Enlitech), where the photocurrent was amplified and modulated by a lock-in instrument. EQE_EL_ measurements were performed by applying external voltage/current sources through the devices (ELCT-3010, Enlitech). All the devices were prepared for EQE_EL_ measurements according to the optimal device fabrication conditions. EQE_EL_ measurements were carried out from 0 to 4 V

**1.13 Materials and Synthesis**

All manipulations involving air-sensitive reagents were performed under an inert atmosphere of dry nitrogen. Compounds PM6, fBDT-Sn and BDD-Br were purchased from Derthon Optoelectronic Materials Science Technology Co. Ltd. (Shenzhen, China). The *M*_n_ of PM6 was determined to be 30.21 kDa, with polydispersity indexes (PDIs) of 1.75. All the other starting materials, unless otherwise specified, were purchased commercially and used as received without further purification.

**Scheme S1** Synthetic routes of the Ptpy-Br monomer and targeted polymers.

*Synthesis of compound* **1**

5-Bromo-2-iodopyridine (3.0 g, 10.6 mmol) and (4-bromophenyl)boronic acid (1.5 g, 7.5 mmol) were added into a mixed solvent (30 mL) of toluene, ethanol, and H_2_O (7:1:2, v/v/v) under a N_2_ atmosphere. Then Pd(PPh_3_)_4_ (0.2 g, 0.2 mmol) and K_2_CO_3_ (2.9 g, 21.2 mmol) were added into the reaction system. The reaction was allowed to proceed at 85 ^o^C for 18 hours. After cooling to room temperature, the mixture was diluted by 100 mL of ethyl acetate (EA) and washed with water. The collected organic solvent was dried over Na_2_SO_4_ and removed under the reduced pressure, followed by purification using the silica gel column chromatography eluting with a mixed solvents of PE/EA (5/1) to afford compound **3** (1.9 g, 81%). ^1^H NMR (400 MHz, CDCl_3_) δ 8.72-8.71 (m, 1H), 7.86-7.82 (m, 3H), 7.59-7.56 (m, 3H).

*Synthesis of compound* **2**

Under a N_2_ atmosphere, compound **1** (1.6 g, 5.2 mmol) and K_2_PtCl_4_ (1.8 g, 4.3 mmol) were allowed to react in a mixture solvent (28 mL) of 2-methoxyethanol and water (3:1, v/v) at 110 ^o^C for 18 hours. Then, the reaction mixture was cooled to room temperature and deionized water was added to precipitate the Pt(II) μ-chloro-bridged dimer (1.6 g, 70%). The solid was filtrated, collected, dried and used for the next step without further purification.

*Synthesis of compound* **4**

Benzaldehyde (2.0 g, 18.8 mmol) was mixed with excess pyrrole (10.1 g, 150 mmol). Then, trifluoroacetic acid (TFA, 11.4 g, 100 mmol) was added with stirring for 2 hours at room temperature under N_2_. The unreacted pyrrole was removed under reduced pressure. The crude product was purified by the silica column chromatography to afford compound **3** (2.3 g, 56%). ^1^H NMR (400 MHz, CDCl_3_) δ 7.92 (s, 2H), 7.35-7.33 (m, 2H), 7.29-7.26 (m, 1H), 7.24-7.21 (m, 2H), 6.70-6.69 (m, 2H), 6.18-6.16 (m, 2H), 5.94-5.92 (m, 2H), 5.48 (s, 1H). Then, the intermediate product was reacted with 2,3-dicyano-5,6-dichlorobenzoquinone (DDQ, 3.6 g, 15.8 mmol) in dichloromethane to give compound **4** (1.9 g, 80%). The activated ligand was filtrated over celite and directly used for the next step without further purification.

*Synthesis of compound* **5**

Compound **2** (1.0 g, 0.9 mmol), compound **4** (0.6 g, 2.7 mmol) and Na_2_CO_3_ (1.0 g, 9.4 mmol) were added to 10 mL dichloroethane (DCE), and the mixture was heated to 80 °C for 12 h. After cooling to room temperature, the mixture was washed with deionized water and the organic layers were removed under reduced pressure. The crude product was purified on a silica gel column using hexane/CH_2_Cl_2_ as the eluent to furnish a red solid (0.3 g, 50%). ^1^H NMR (400 MHz, CDCl_3_) δ 8.72-8.71 (d, 1H), 8.01-7.99 (m, 2 H), 7.79 (1 H, s), 7.60-7.46 (m, 7 H), 7.40-7.38 (d, 1 H), 7.32-7.29 (m, 1 H), 6.89-6.88 (m, 1 H), 6.74-6.72 (m, 1 H), 6.63-6.61 (m, 1 H), 6.54-6.52 (m, 1 H). ^13^C NMR (101 MHz, CDCl_3_) δ 166.36, 152.81, 151.46, 150.66, 148.10, 144.33, 140.77, 137.91, 137.84, 137.48, 136.86, 136.80, 131.85, 130.70, 130.51, 128.93, 127.61, 127.42, 124.58, 116.23. MS (MALDI-TOF) [m/z]: found [M]^+^ 726.942, calculated 726.941 (C_26_H_17_N_3_Br_2_Pt).

*General synthetic procedure of the polymers*: fBDT-Sn (188.5 mg, 0.2 mmol) and bromide monomers of BDD-Br and Ptpy-Br at varied ratios (0.99/0.01, 0.97/0.03, 0.95/0.05) were dissolved in dry toluene (5 mL), and Pd_2_(dba)_3_ (5.5 mg, 0.006 mmol) and P-(o-toly)_3_ (14.60 mg, 0.048 mmol) was added to the mixture being flushed with argon for five minutes. Then, the reaction mixture was purged with argon for another 15 min and stirred at 110 °C for 2 days. After the reaction was completed, the reaction mixture was cooled down to room temperature and precipitated into methanol (200 mL). The crude polymer was subjected to Soxhlet extractions with acetone, methanol and hexane to remove the impurities and oligomers. Then the chloroform fraction was concentrated, and the solution was filtered through a silica gel column.

**PM6-Pt1**: fBDT-Sn (188.5 mg, 0.2 mmol), BDD-Br (151.8 mg, 0.198 mmol), Ptpy-Br (1.5 mg, 0.002 mmol). Yield (185 mg, 86%). Elemental analysis calcd (%) for PM6-Pt1: N, 0.034%; C, 66.72%; H, 6.36%; S, 20.85%. Found: N, 0.04%; C, 66.32%; H, 6.54%; S, 20.46%

**PM6-Pt3**: fBDT-Sn (188.5 mg, 0.2 mmol), BDD-Br (148.7 mg, 0.194 mmol), Ptpy-Br (4.5 mg, 0.006 mmol). Yield (185 mg, 76%). Elemental analysis calcd (%) for PM6-Pt3: N, 0.10%; C, 66.61%; H, 6.33%; S, 20.65%. Found: N, 0.08%; C, 66.30%; H, 6.50%; S, 20.51%

**PM6-Pt5**: fBDT-Sn (188.5 mg, 0.2 mmol), BDD-Br (145.6 mg, 0.190 mmol), Ptpy-Br (7.5 mg, 0.01 mmol). Yield (175 mg, 72%). Elemental analysis calcd (%) for PM6-Pt5: N, 0.17%; C, 66.50%; H, 6.30%; S, 20.46%. Found: N, 0.12%; C, 66.24%; H, 6.48%; S, 20.55%

**1.14 Supporting Figures and Tables**


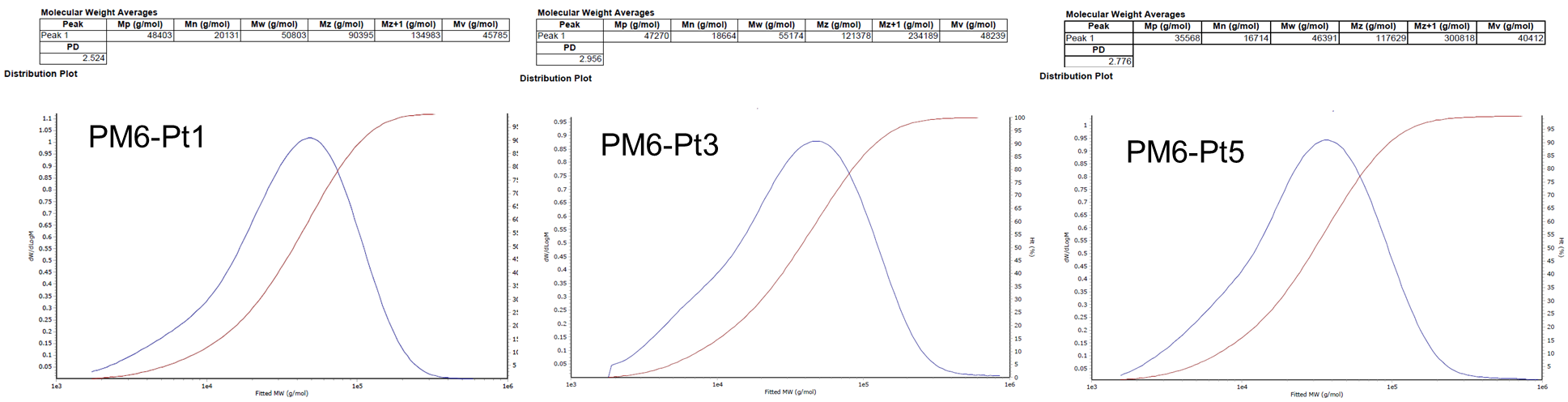


**Figure S1**. GPC traces of the polymers measured with 1,2,4-trichlorobenzene as the eluent and polystyrene as a standard at 140 ℃

**Table S1** The Pt amount tested from the ICP-OES

| Polymer | Sample Weight | Constant Volume | Detected  Element | Test  Result | Pt content  (ug/mg) |
| --- | --- | --- | --- | --- | --- |
| PM6-Pt1 | 0.4822 mg | 10 mL | Pt | 0.0772 mg/L | 1.601 |
| PM6-Pt3 | 0.4765 mg | 10 mL | Pt | 0.2303 mg/L | 4.834 |
| PM6-Pt5 | 0.5021 mg | 10 mL | Pt | 0.4029 mg/L | 8.026 |

**
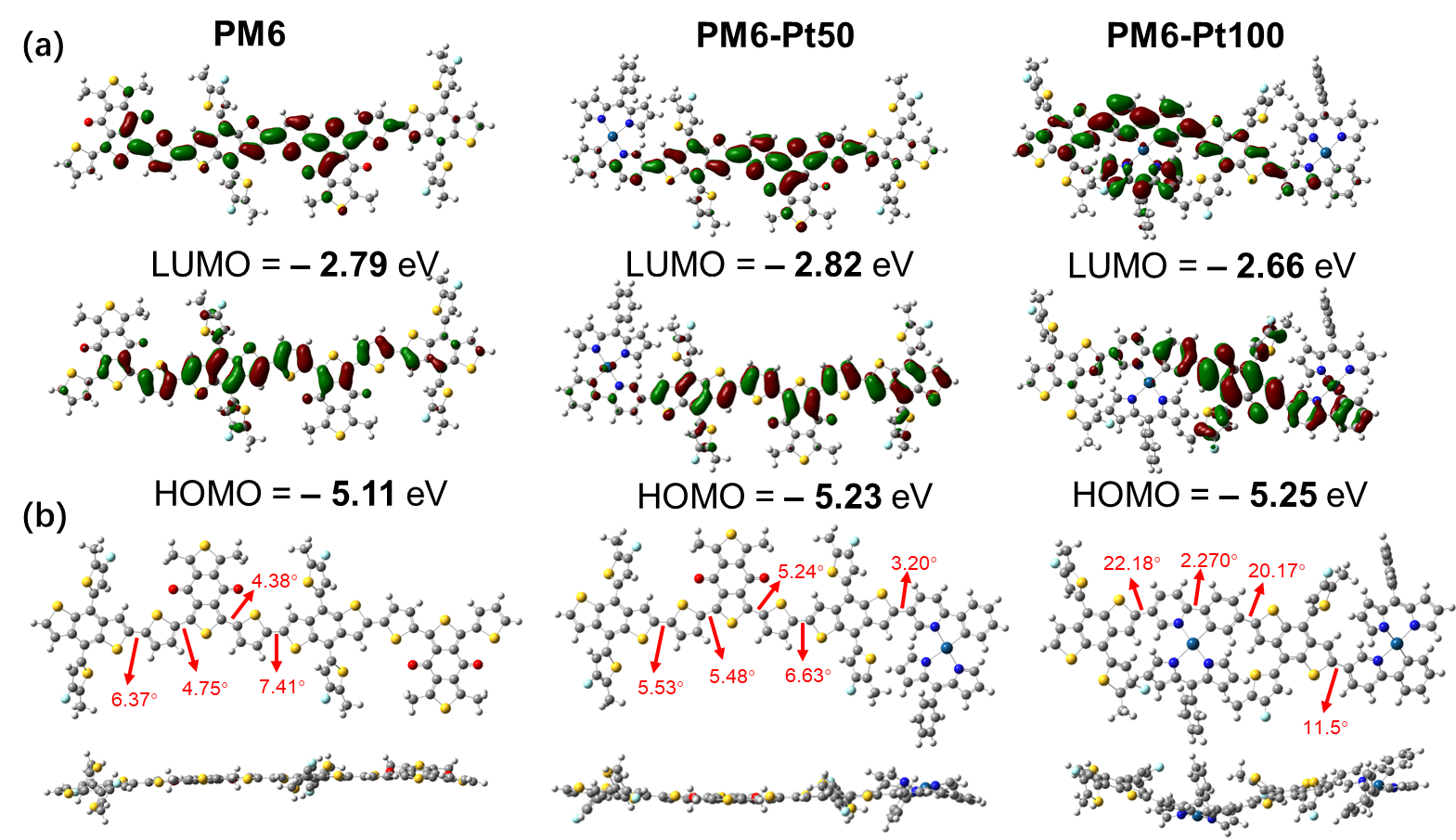
**

**Figure S2**. (a) Calculated energy levels and (b) calculated geometries of PM6, PM6-Pt50, and PM6-Pt100.

**Figure S3**. UV–Vis absorption spectra of PM6-Pt1, PM6-Pt3 and PM6-Pt5 in dilute chloroform solution.


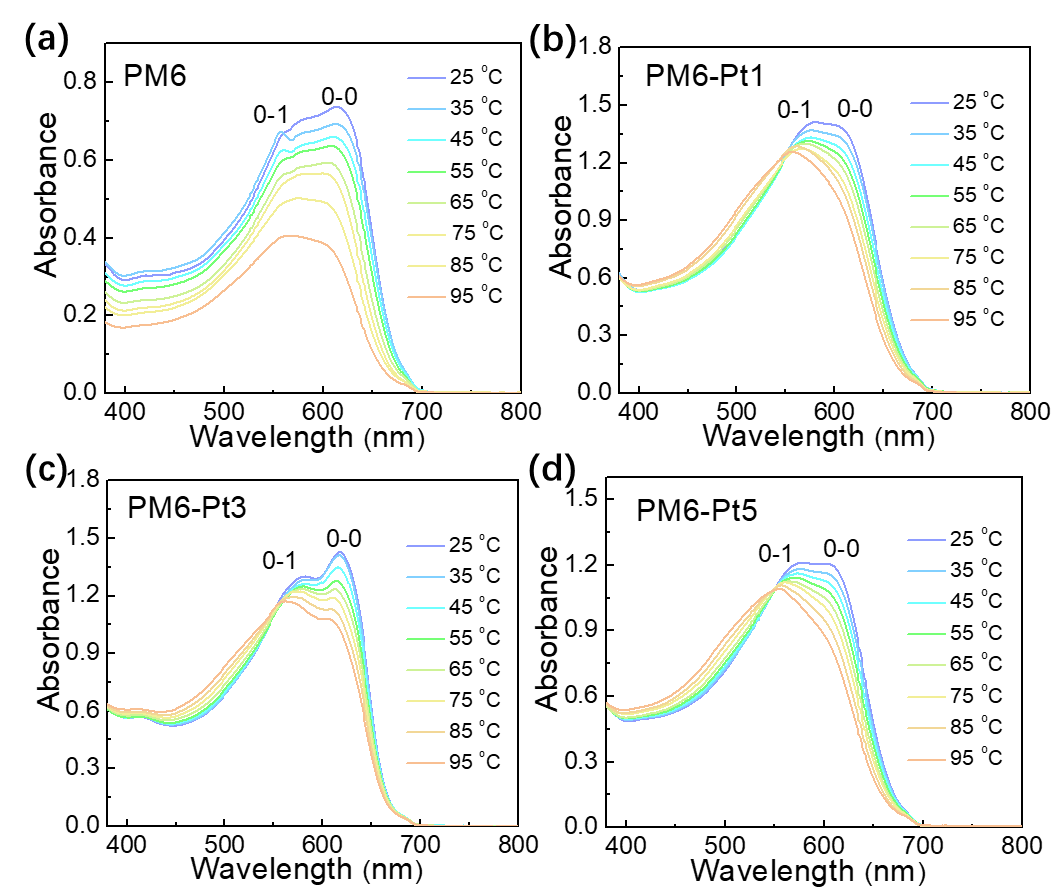


**Figure S4**. The temperature-dependent UV-Vis absorption spectra of the polymers.

**Figure S5**. Plot of intensity ratio between *λ*_0–0_ and *λ*_0–1_ versus solution temperature of the polymers.


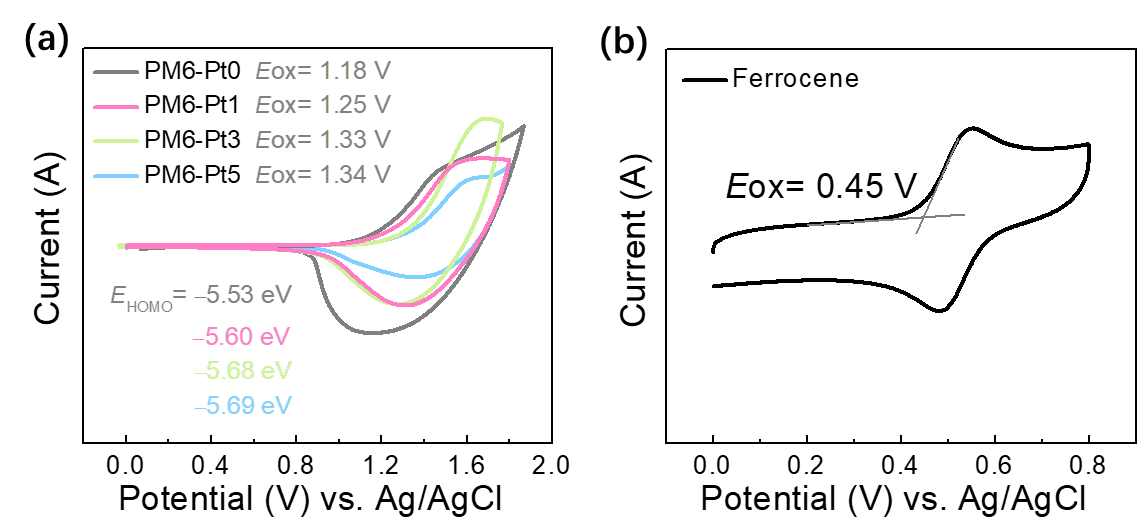


**Figure S6**. CV curves of (a) the polymers and (b) ferrocene.

**Figure S7**. TRPL decay spectra of PM6, PM6-Pt1, PM6-Pt3 and PM6-Pt5 films.

**Table S2** Detailed parameters of TRPL of the pure polymer films were fitted by a biexponential function: *i* = A_1_ exp(−t/τ_1_) + A_2_ exp(−t/τ_2_), with two lifetimes of *τ*_1_ and *τ*_2_ and prefactors of *A*_1_ and *A*_2_.

| Polymer film | *A*_1_ | *τ*_1_ (ps) | *A*_2_ | *τ*_2_ (ps) | *τ*_m_ (ps)^a)^ |
| --- | --- | --- | --- | --- | --- |
| PM6 | 0.99 | 0.239 | 0.01 | 1.833 | 0.351 |
| PM6-Pt1 | 0.91 | 0.251 | 0.09 | 1.875 | 0.943 |
| PM6-Pt3 | 0.89 | 0.262 | 0.11 | 1.878 | 1.022 |
| PM6-Pt5 | 0.87 | 0.267 | 0.13 | 1.879 | 1.094 |

^a)^ The average lifetime values were extracted by a biexponential function fit and calculated according to the equation: *τ*_m_ = (*A*_1_*τ*_1_^2^ + *A*_2_*τ*_2_^2^)/(*A*_1_*τ*_1_ + *A*_2_*τ*_2_)

**
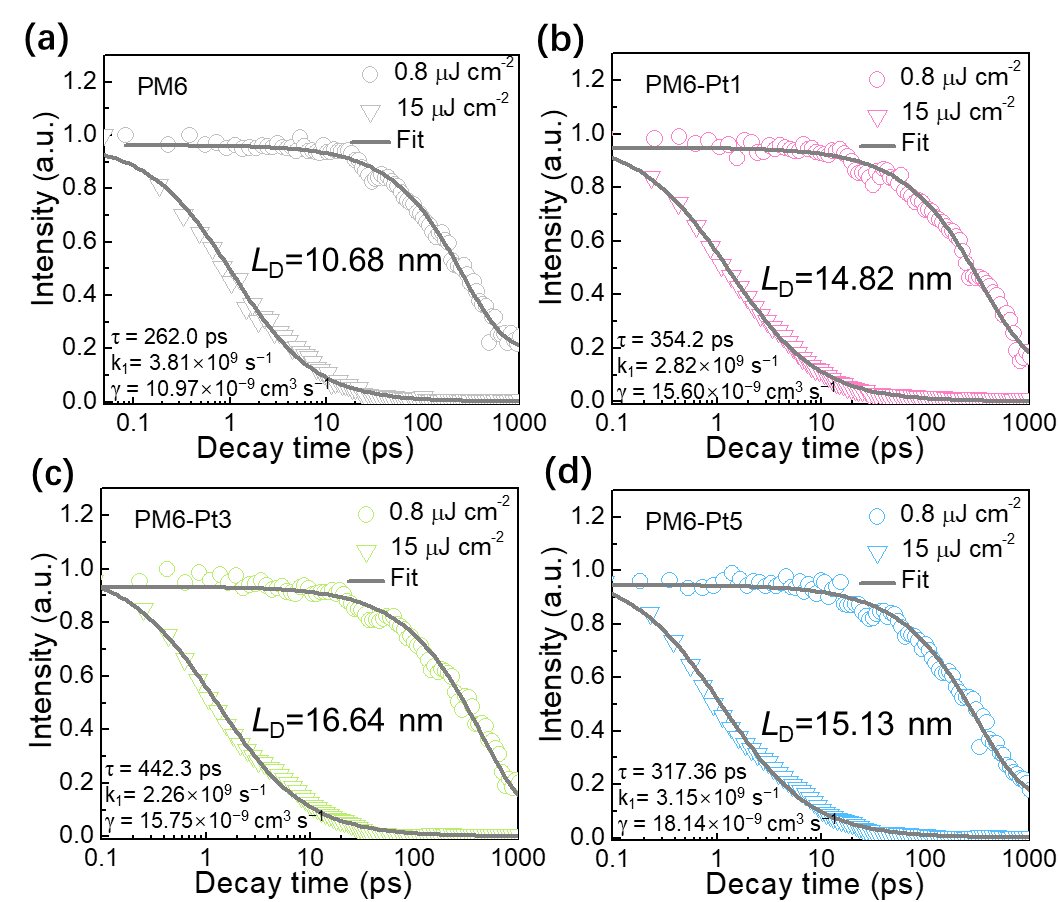
**

**Figure S8**. Decay dynamics of excitons of PM6 (a), PM6-Pt1 (b), PM6-Pt3 (c), and PM6-Pt5 (d) films under 500 nm pump with power fluxes of 0.8 and 15 μJ cm^−2^.

**Table S3** Fitting parameters for the pump fluence-dependent TA decay curves.

| Film | Pump energy  (μJ cm^−2^) | *τ*  (ps) | *k*  (×10^9^ s^−1^) | *γ*  (×10^−9^ cm^3^ s^−1^) | *D*  (×10^−3^ cm^2^ s^−1^) | *L*_D_  (nm) |
| --- | --- | --- | --- | --- | --- | --- |
| PM6 | 0.8 | 262.06 | 3.81 | / | / | / |
|  | 15 | / | / | 10.97 | 4.36 | 10.68 |
| PM6-Pt1 | 0.8 | 354.21 | 2.82 | / | / | / |
|  | 15 | / | / | 15.60 | 6.21 | 14.82 |
| PM6-Pt3 | 0.8 | 442.37 | 2.26 | / | / |  |
|  | 1.5 | / | / | 15.75 | 6.26 | 16.64 |
| PM6-Pt5 | 0.8 | 317.36 | 3.15 | / | / |  |
|  | 1.5 | / | / | 18.14 | 7.22 | 15.13 |

**Figure S9**. The *J*^1/2^−*V* characteristics of the hole-only devices based on the pure polymer films.

**Table S4** Hole mobility in single-carrier devices for the pure polymer films.

| Film | *μ*_h_  (cm^2^ V^−1^ s^−1^) |
| --- | --- |
| PM6 | 2.24 × 10^−4^ |
| PM6-Pt1 | 1.68 × 10^−4^ |
| PM6-Pt3 | 1.61 × 10^−4^ |
| PM6-Pt5 | 1.58 × 10^−4^ |

**
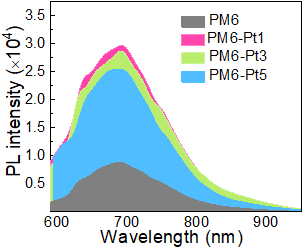
**

**Figure S10**. PL spectra of the pure polymer films under 532 nm laser excitation.


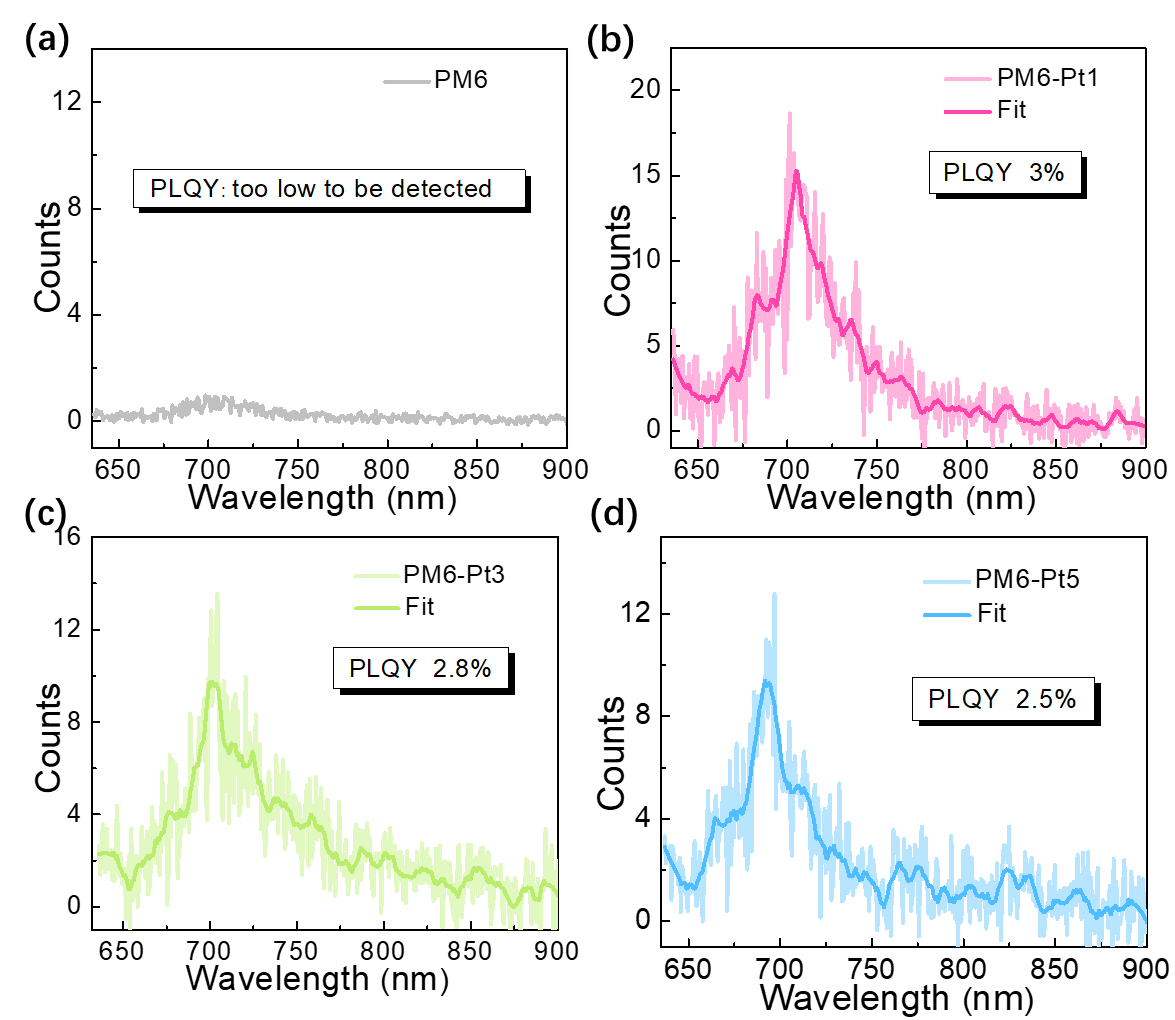


**Figure S11**. PLQY measurement results of (a) PM6, (b) PM6-Pt1, (c) PM6-Pt3 and (d) PM6-Pt5.

**Table S5** Summary of photophysical properties and rate constants of the polymer films.

| Polymer | λ_em_  (nm) | *Ф*_PL_  (%) | *K*_P_  (×10^9^ s^−1^) | *K*_d_  (×10^8^ s^−1^) | *k*_r, s_  (×10^7^ s^−1^) | *k*_nr, s_  (×10^9^ s^−1^) | *k*_ISC_  (×10^8^ s^−1^) |
| --- | --- | --- | --- | --- | --- | --- | --- |
| PM6 | 600 | / | 4.22 | 5.45 | / | / | / |
| PM6-Pt1 | 600 | 3 | 3.98 | 5.33 | 11.1 | 3.55 | 3.2 |
| PM6-Pt3 | 600 | 2.8 | 3.81 | 5.32 | 9.65 | 3.34 | 3.74 |
| PM6-Pt5 | 600 | 2.5 | 3.74 | 5.32 | 8.29 | 3.23 | 4.27 |

**Table S6** Morphology data of pure films.

| Film | in plane (IP) | | | | out of plane (OOP) | | | |
| --- | --- | --- | --- | --- | --- | --- | --- | --- |
|  | Position (Å^-1^) | d-spacing*^a^*  Å | FWHM  Å | CCL*^b^*  Å | Position (Å^-1^) | d-spacing*^a^*  Å | FWHM  Å | CCL*^b^*  Å |
| PM6 | 0.289 | 21.73 | 0.117 | 48.30 | 1.680 | 3.73 | 0.408 | 13.85 |
| PM6-Pt1 | 0.291 | 21.58 | 0.124 | 45.58 | 1.685 | 3.72 | 0.412 | 13.71 |
| PM6-Pt3 | 0.291 | 21.58 | 0.131 | 43.14 | 1.686 | 3.72 | 0.413 | 13.68 |
| PM6-Pt5 | 0.291 | 21.58 | 0.142 | 39.80 | 1.687 | 3.72 | 0.415 | 13.61 |

*^a^* Obtained using the equation of *d* = 2π/*q*, in which *q* is the corresponding *x*-coordinate of the diffraction peak. *^b^* Calculated using the equation: CCL= 2π*K*/*w*, in which *w* is the full width at half maximum and *K* is a form factor.

**Figure S12**. The Raman spectra of pure polymer films.


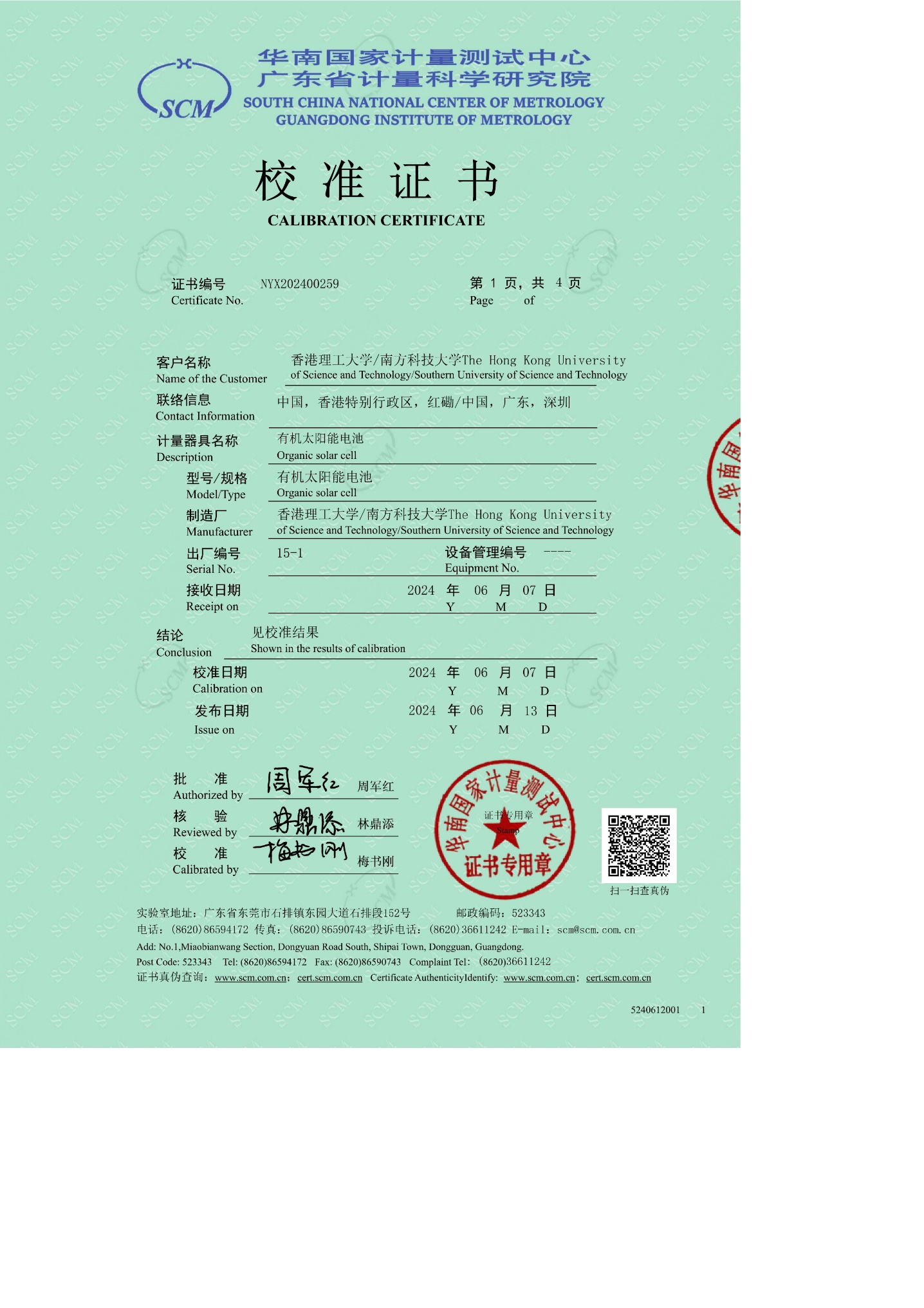


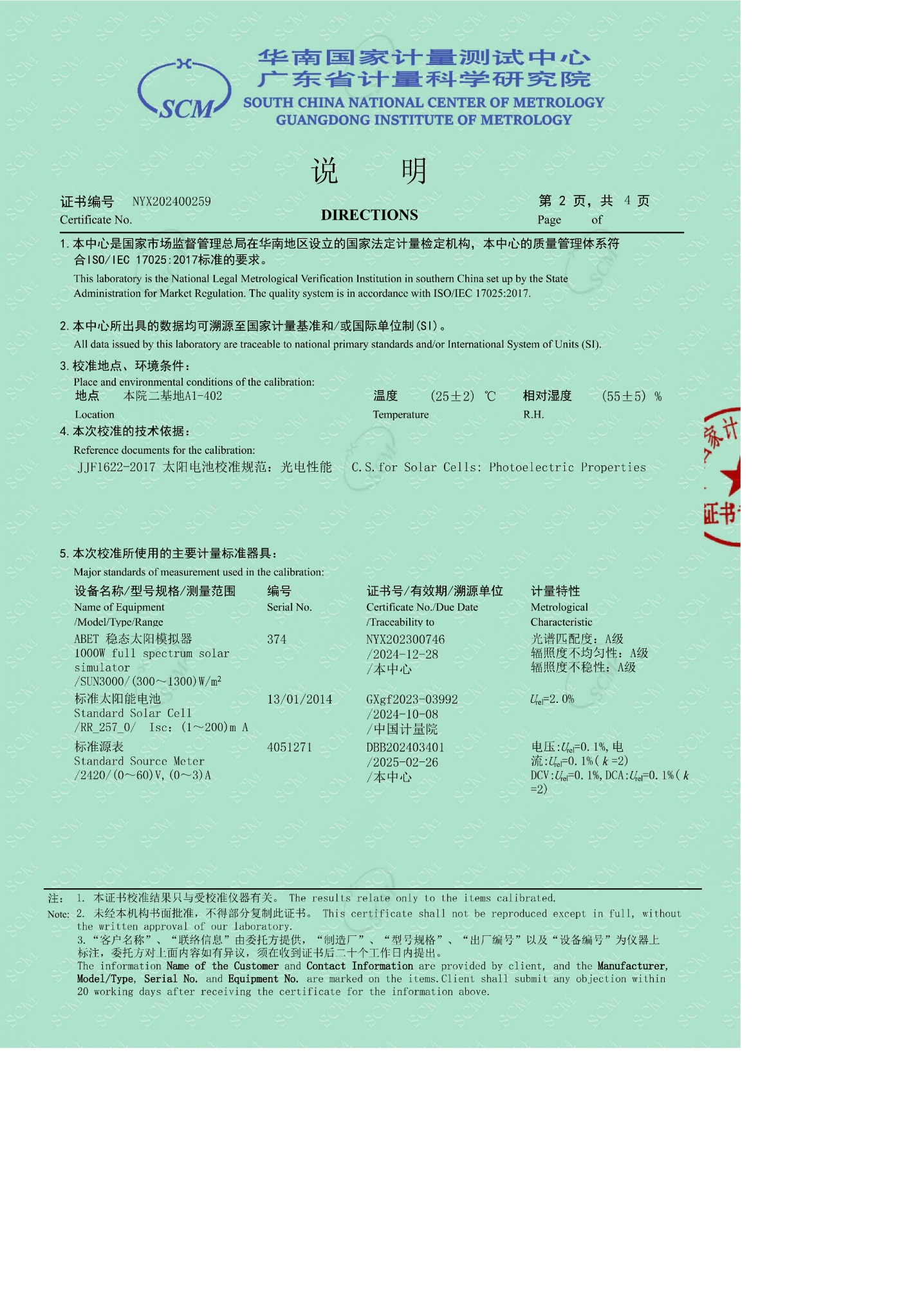


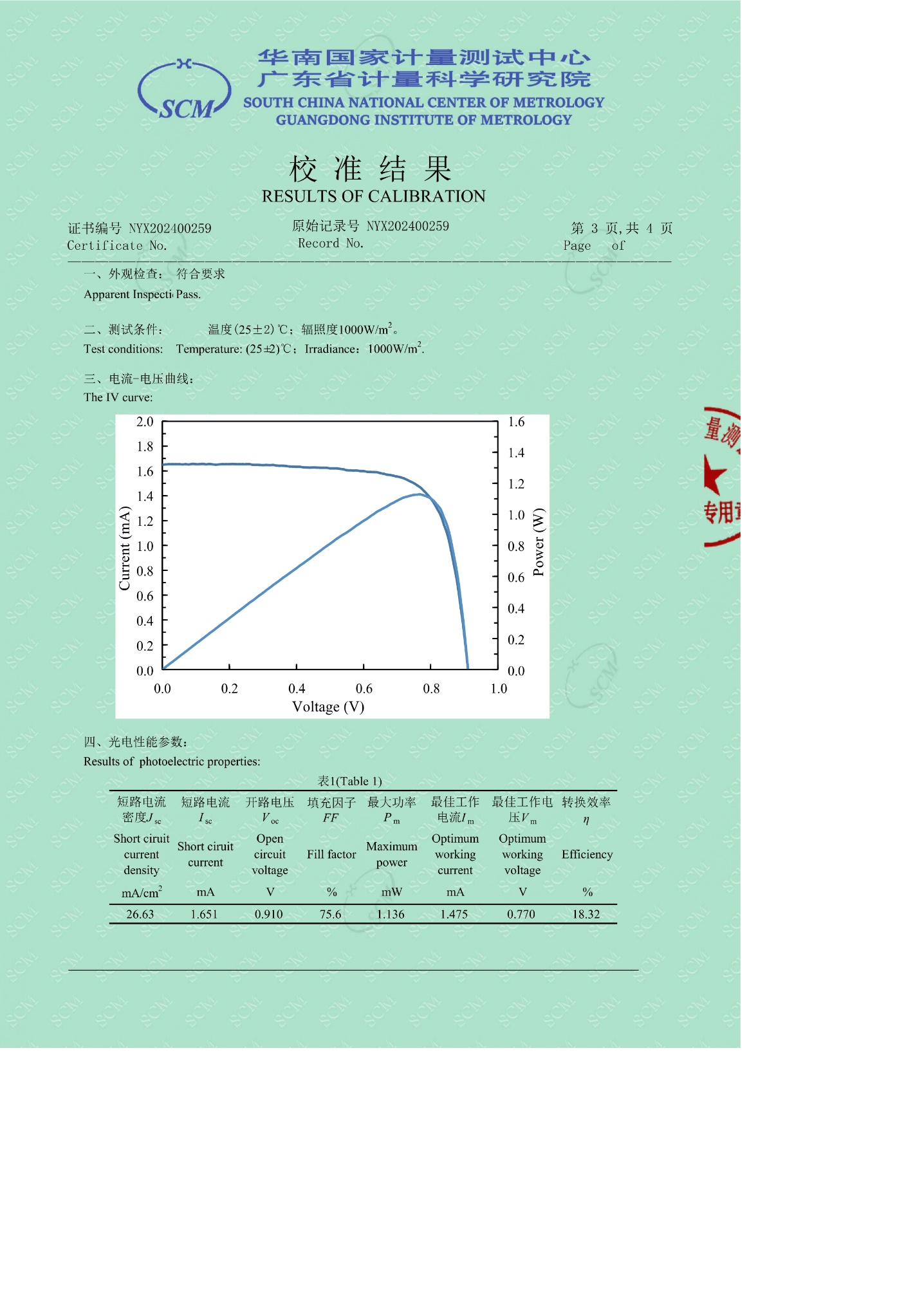


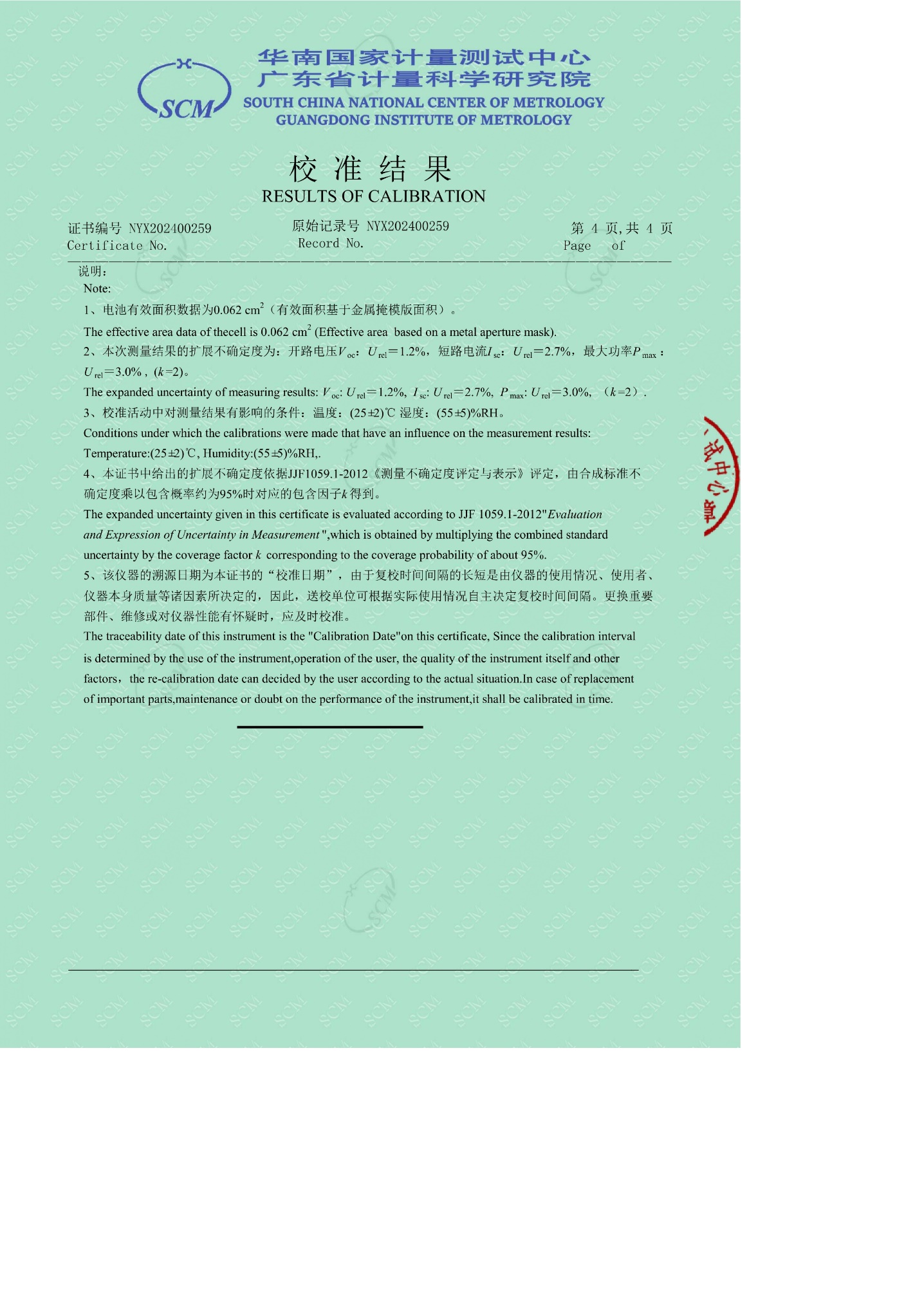


**Figure S13**. Certified efficiency of PM6-Pt1:L8-BO binary system.

**Figure S14**. UV–Vis absorption spectra of the blend films.

**
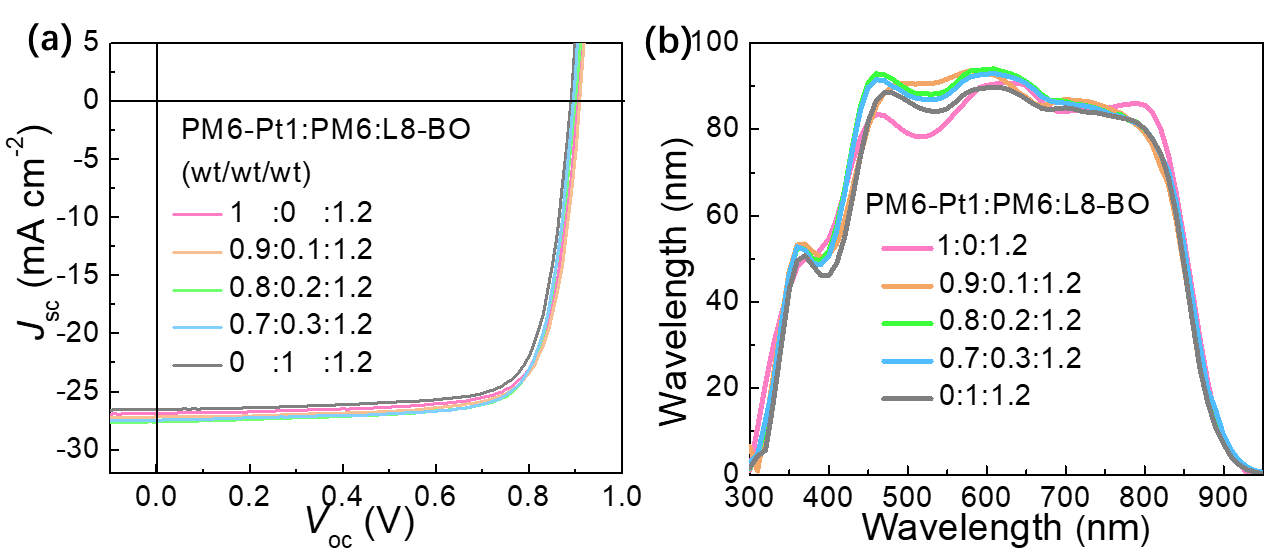
**

**Figure S15**. (a) *J*−*V* characteristics of the ternary OSCs based on PM6-Pt1:PM6:L8-BO with different weight ratio under AM1.5G illumination (100 mW cm^−2^). (b) EQE spectra of the corresponding devices.

**Table S7** Photovoltaic parameters of OSCs based on PM6-Pt:PM6:L8-BO blended films with different weight ratios.

| PM6-Pt1:PM6:L8-BO  (wt/wt/wt) | *V*_oc_ | *J*_sc_ | *J*_sc_ | FF | PCE |
| --- | --- | --- | --- | --- | --- |
|  | (V) | (mA cm^-2^) | (mA cm^−2^)^a)^ | (%) | (%)^b)^ |
| 1:0:1.2 | 0.91 | 26.87 | 25.79 | 75.83 | 18.54 (18.35$\pm$0.19) |
| 0.9:0.1:1.2 | 0.91 | 27.13 | 26.04 | 76.75 | 18.94 (18.60$\pm$0.34) |
| 0.8:0.2:1.2 | 0.90 | 27.56 | 26.45 | 77.60 | 19.24 (18.92$\pm$0.32) |
| 0.7:0.3:1.2 | 0.89 | 27.41 | 26.31 | 76.97 | 18.77 (18.61$\pm$0.10) |
| 0:1:1.2 | 0.88 | 26.41 | 25.35 | 77.88 | 18.10 (18.00$\pm$0.10) |

^a)^ Calculated current densities from EQE curves. ^b)^ Average PCEs from ten devices.

**Table S8** The parameters extracted from photo-CELIV.

| Active layer | *A*  (V ms ^–1^) | *t_max_*  (μs) | *Δj* (mA) | *j* (0) | *Δj/j*(0) | *d*  (nm) | *μ*  (cm^2^ V^–1^ s^–1^) |
| --- | --- | --- | --- | --- | --- | --- | --- |
| PM6:L8-BO | 50 | 3.03 | 0.263 | 0.150 | 1.750 | 115 | 1.17 × 10^-4^ |
| PM6-Pt1:L8-BO | 50 | 2.76 | 0.258 | 0.144 | 1.791 | 115 | 1.40 × 10^-4^ |
| PM6-Pt3:L8-BO | 50 | 3.15 | 0.273 | 0.147 | 1.857 | 115 | 1.06 × 10^-4^ |
| PM6-Pt5:L8-BO | 50 | 3.06 | 0.310 | 0.121 | 2.561 | 115 | 9.79 × 10^-5^ |


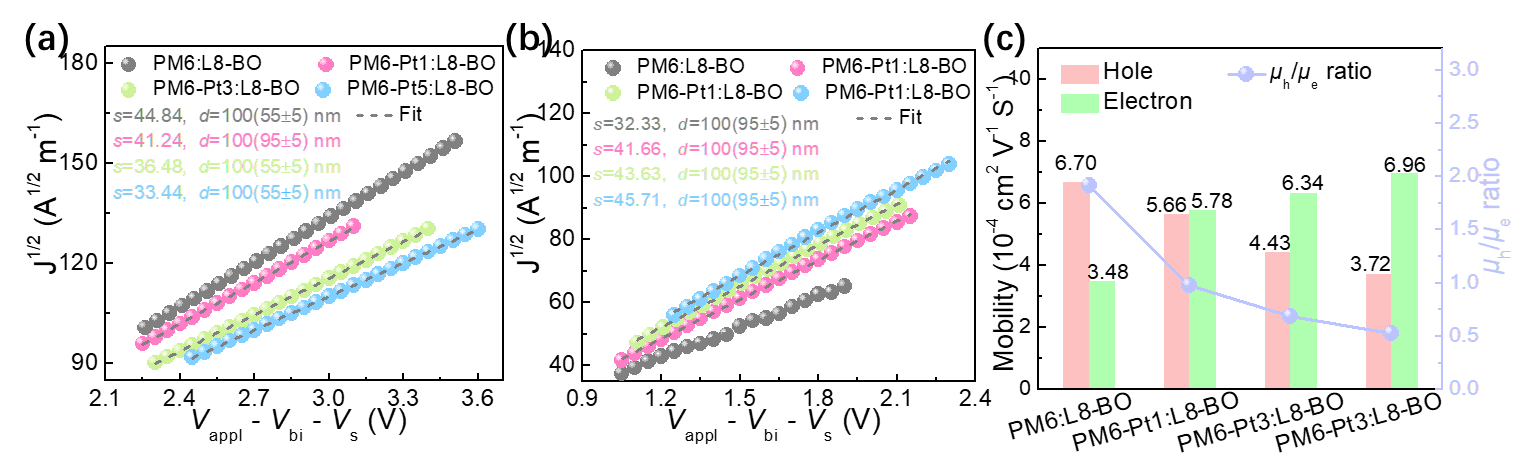


**Figure S16**. The *J*^1/2^−*V* characteristics of hole-only (a) and electron-only (b) devices based on the blend films. (c) The hole and electron mobilities and *μ*_h_/*μ*_e_ ratio of the blend films.

**Table S9** Hole and electron mobility in single-carrier devices for the binary blend films.

| Blend films | *μ*_h_  (cm^2^ V^−1^ s^−1^) | *μ*_e_  (cm^2^ V^−1^ s^−1^) | *μ*_h_ */ μ*_e_ ratio |
| --- | --- | --- | --- |
| PM6:L8-BO | 6.70 × 10^−4^ | 3.48 × 10^−4^ | 1.92 |
| PM6-Pt1:L8-BO | 5.66 × 10^−4^ | 5.78 × 10^−4^ | 0.98 |
| PM6-Pt3:L8-BO | 4.43 × 10^−4^ | 6.34 × 10^−4^ | 0.69 |
| PM6-Pt5:L8-BO | 3.72 × 10^−4^ | 6.96 × 10^−4^ | 0.53 |

**
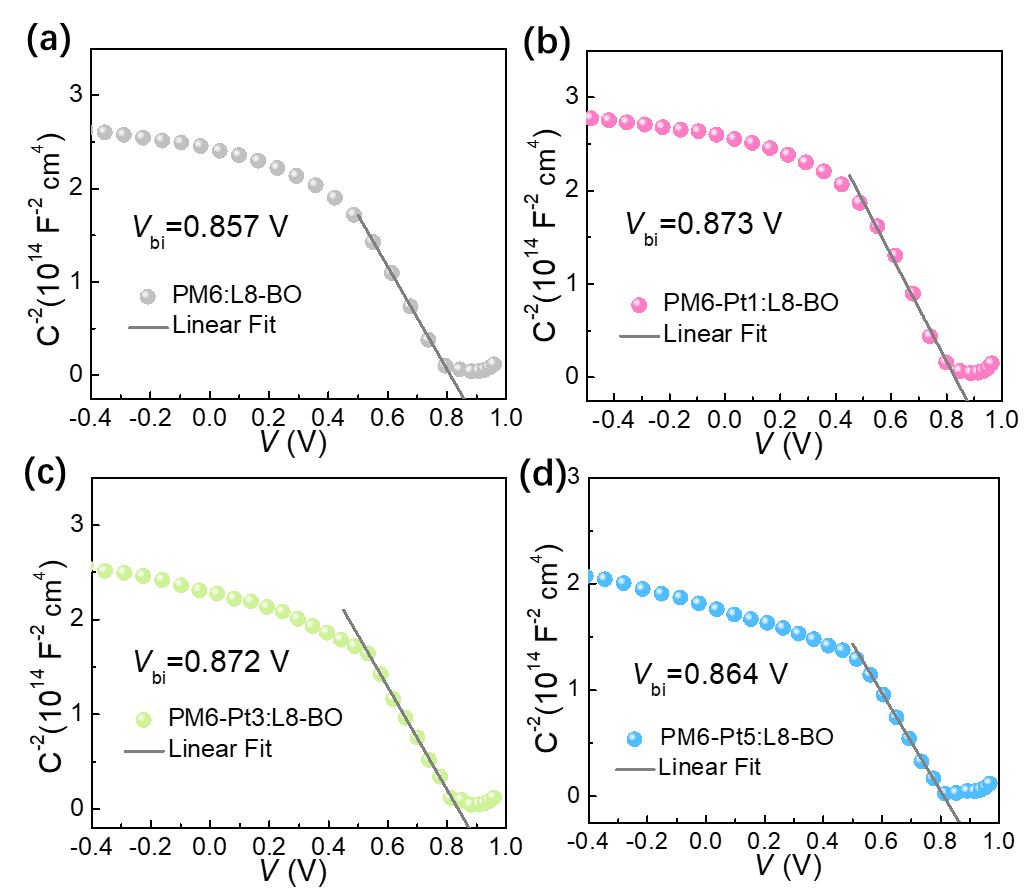
**

**Figure S17**. Mott-Schottky plots for devices based on (a) PM6:L8-BO, (b) PM6-Pt1:L8-BO, (c) PM6-Pt3:L8-BO, and (d) PM6-Pt5:L8-BO.


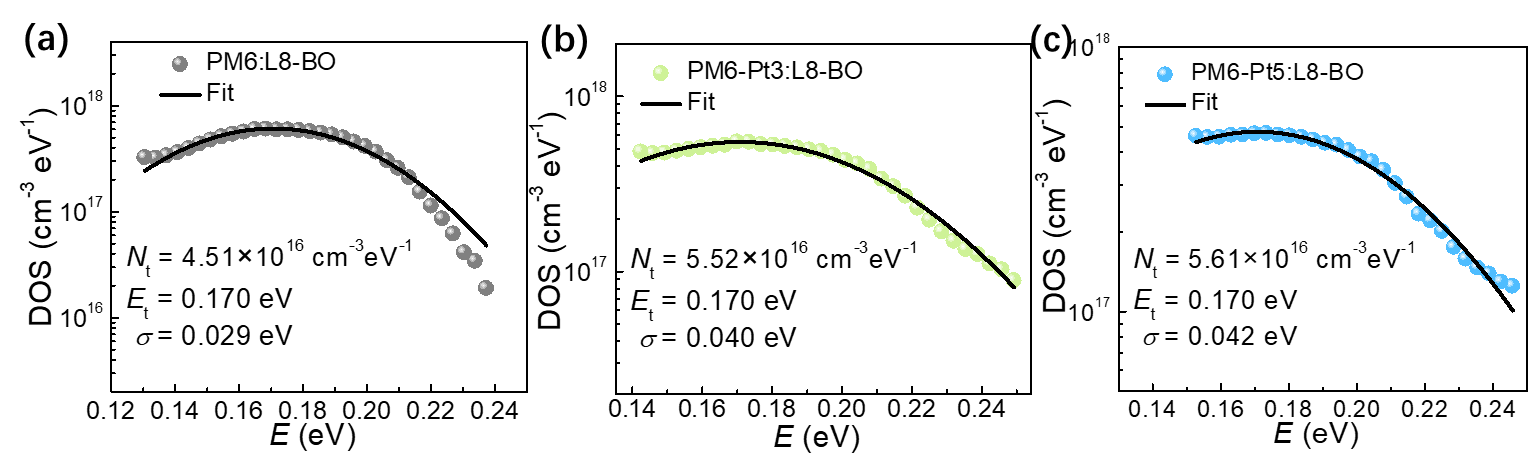


**Figure S18**. Trap density of states (tDOS) spectra and the corresponding Gaussian fitting (solid line)

for devices based on (a) PM6:L8-BO, (b) PM6-Pt3:L8-BO and (b) PM6-Pt5:L8-BO.

**
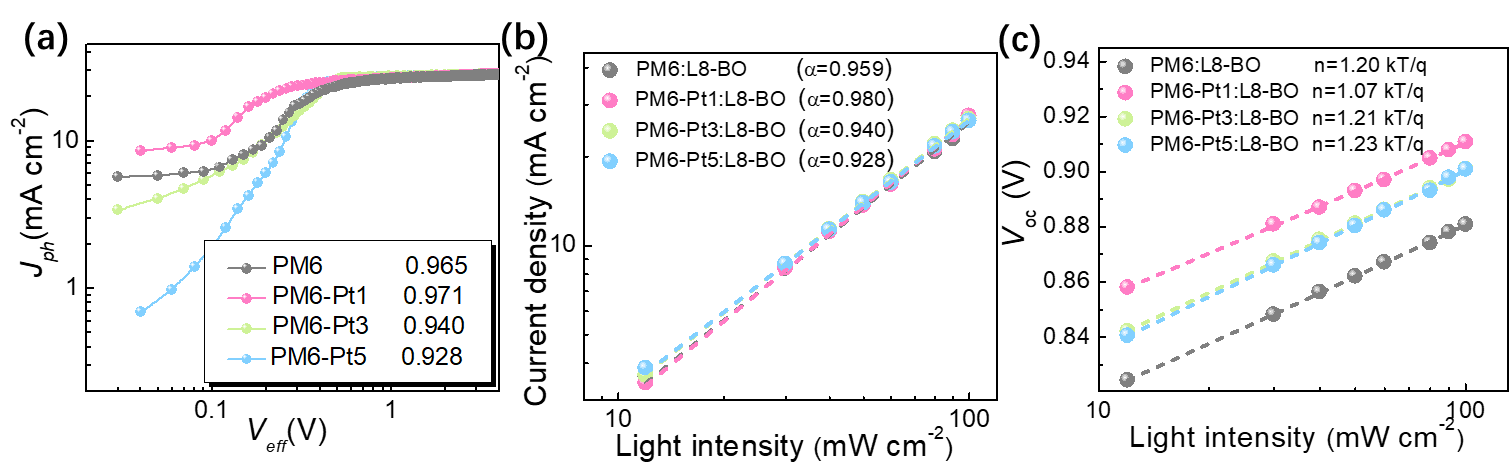
**

**Figure S19**. (a) *J*_ph_ versus *V*_eff_ curves. (b) Light intensity dependence of *J*_sc_. (c) Light intensity dependence of *V*_oc._

**
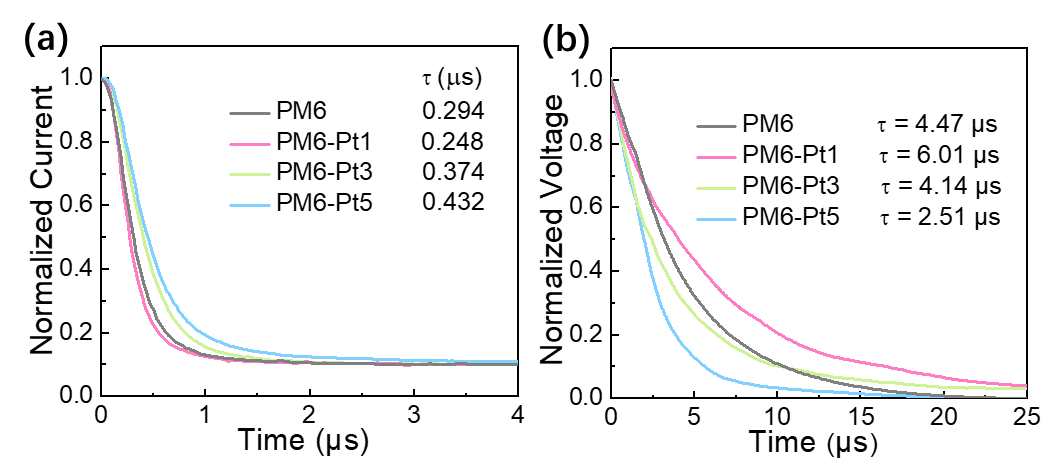
**

**Figure S20**. (a) TPC and (b) TPV measurements of the photovoltaic devices.

**
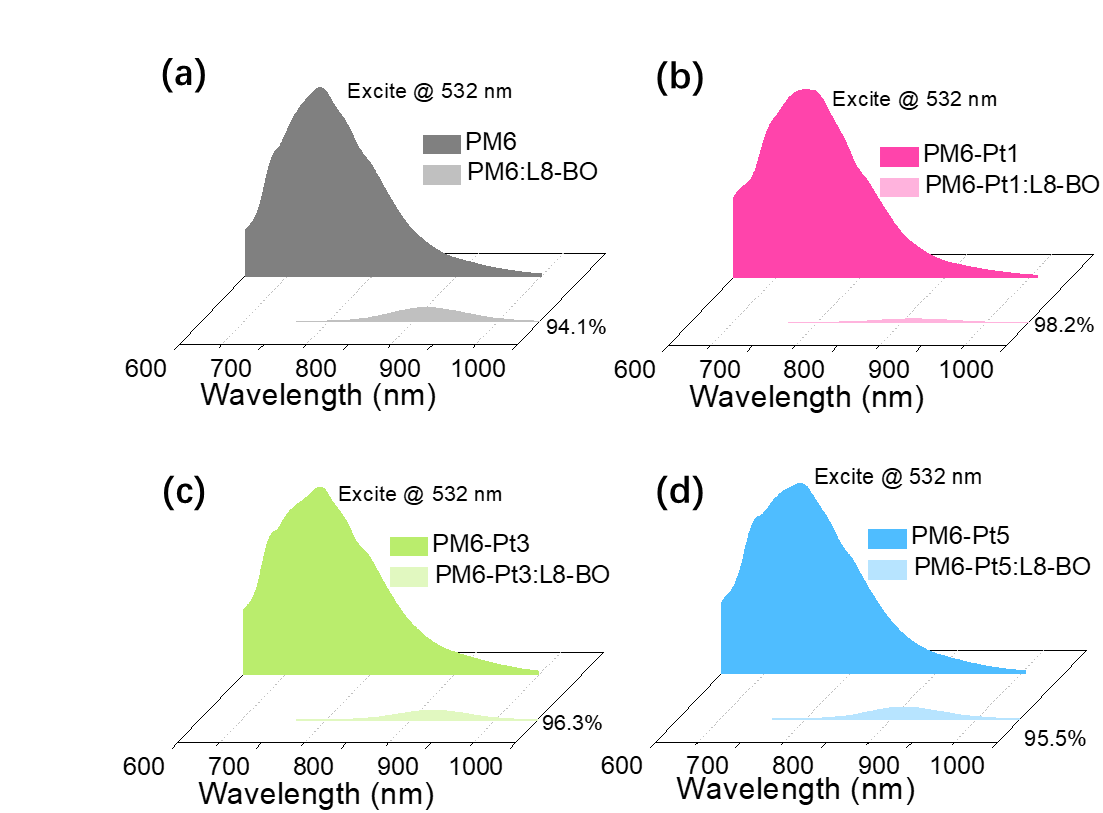
**

**Figure S21**. PL spectra of the pure and binary blend films under 532 nm light excitation.

**
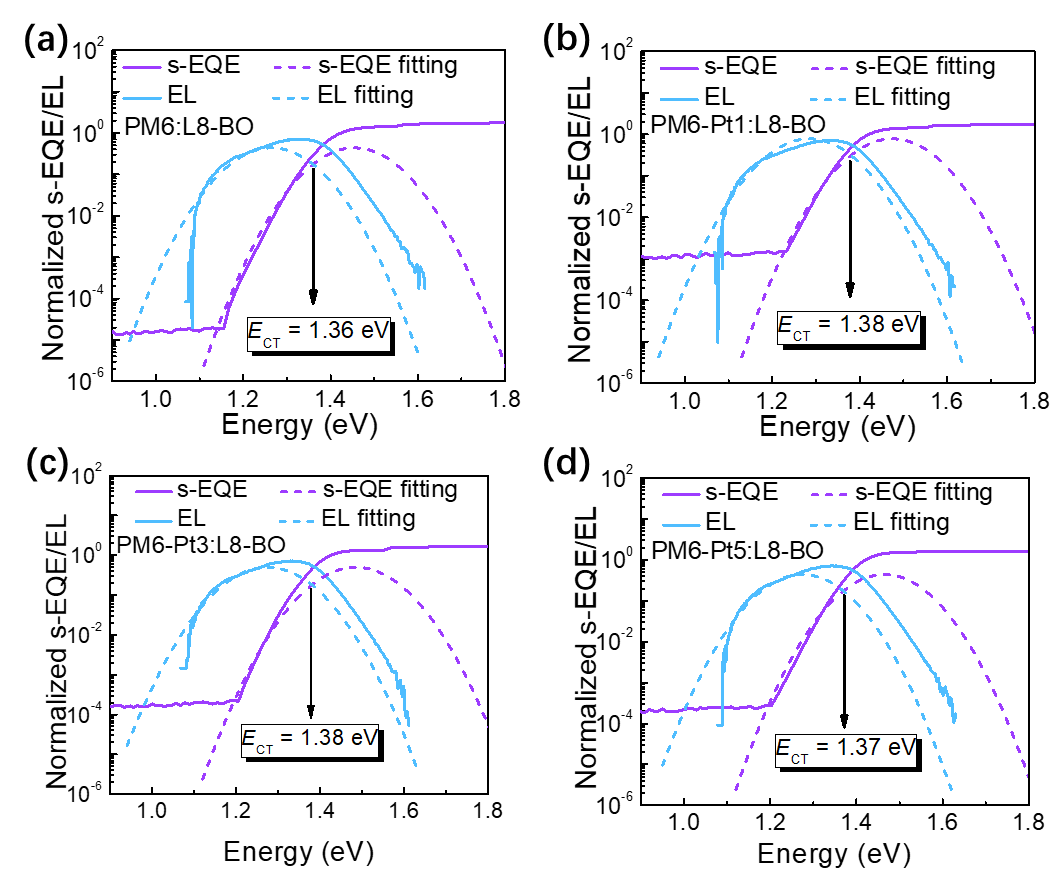
**

**Figure S22**. EL and high-sensitivity EQE spectra of the OSCs for determining E_CT_s of PM6:L8-BO (a), PM6-Pt1:L8-BO (b), PM6-Pt3:L8-BO (c) and PM6-Pt5:L8-BO (d).

**
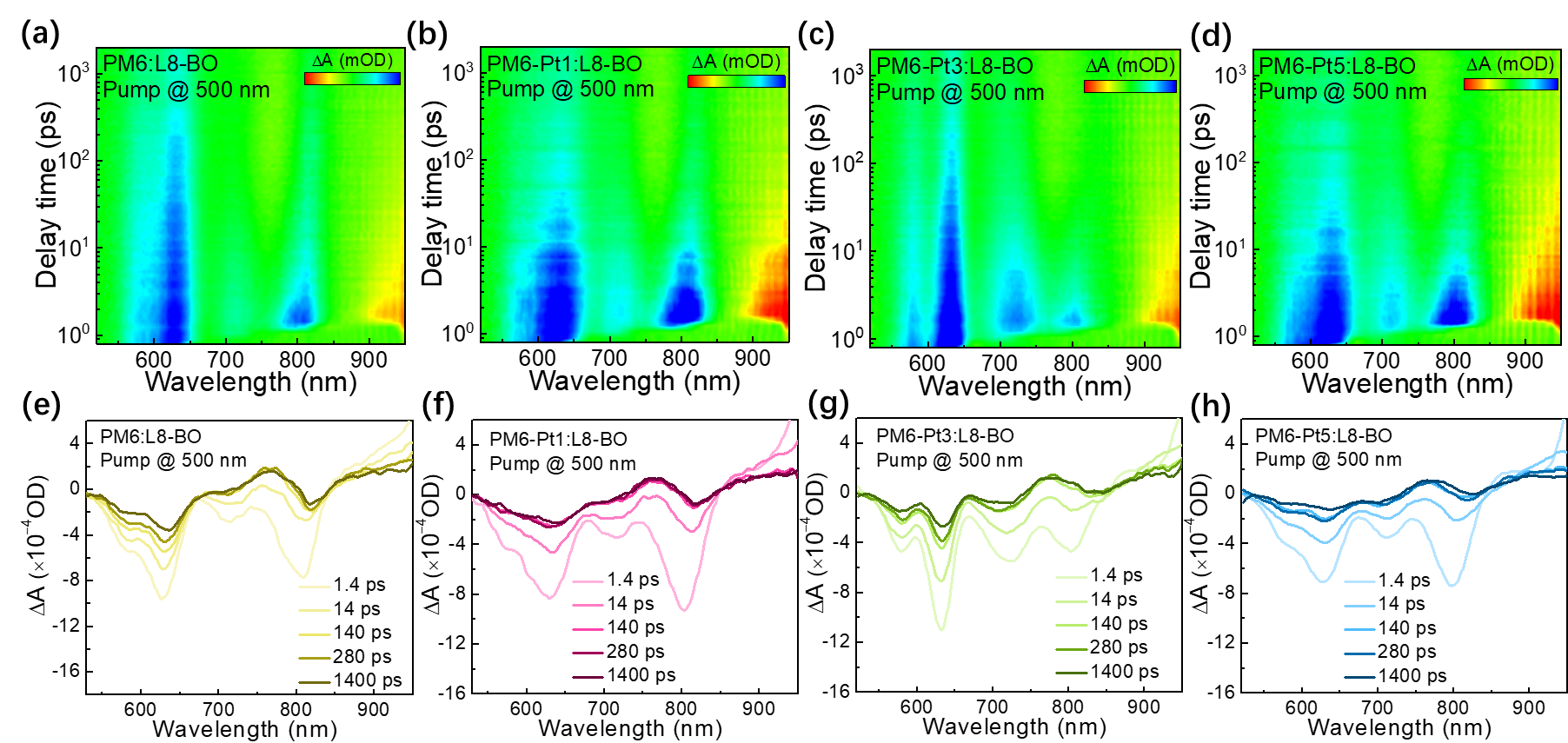
**

**Figure S23**. (a-d) Color plot of TA spectra of PM6:L8-BO, PM6-Pt1:L8-BO, PM6-Pt3:L8-BO and PM6-Pt5:L8-BO films under 500 nm excitation. (e-h) Representative TA spectra of PM6:L8-BO, PM6-Pt1:L8-BO, PM6-Pt3:L8-BO and PM6-Pt5:L8-BO films under 500 nm excitation.

**Table S10** Detailed parameters of TA spectra, the electron transfer kinetics (excited at 500 nm, probe at 625 nm for the blend films were fitted by a biexponential function: *i* = A_1_ exp(−t/τ_1_) + A_2_ exp(−t/τ_2_), with two lifetimes of *τ*_1_ and *τ*_2_ and prefactors of *A*_1_ and *A*_2_.

| Blend film | *A*_1_ | *τ*_1_ (ps) | *A*_2_ | *τ*_2_ (ps) | *τ*_m_ (ps)^a)^ |
| --- | --- | --- | --- | --- | --- |
| PM6:L8-BO | 0.50 | 14.05 | 0.50 | 14.05 | 14.05 |
| PM6-Pt1:L8-BO | 0.50 | 10.02 | 0.50 | 10.02 | 10.02 |
| PM6-Pt3:L8-BO | 0.50 | 13.69 | 0.50 | 13.69 | 13.69 |
| PM6-Pt5:L8-BO | 0.50 | 13.46 | 0.50 | 13.46 | 13.46 |

^a)^ The average lifetime values were extracted by a biexponential function fit and calculated according to the equation: *τ*_m_ = (*A*_1_*τ*_1_^2^ + *A*_2_*τ*_2_^2^)/(*A*_1_*τ*_1_ + *A*_2_*τ*_2_)

**
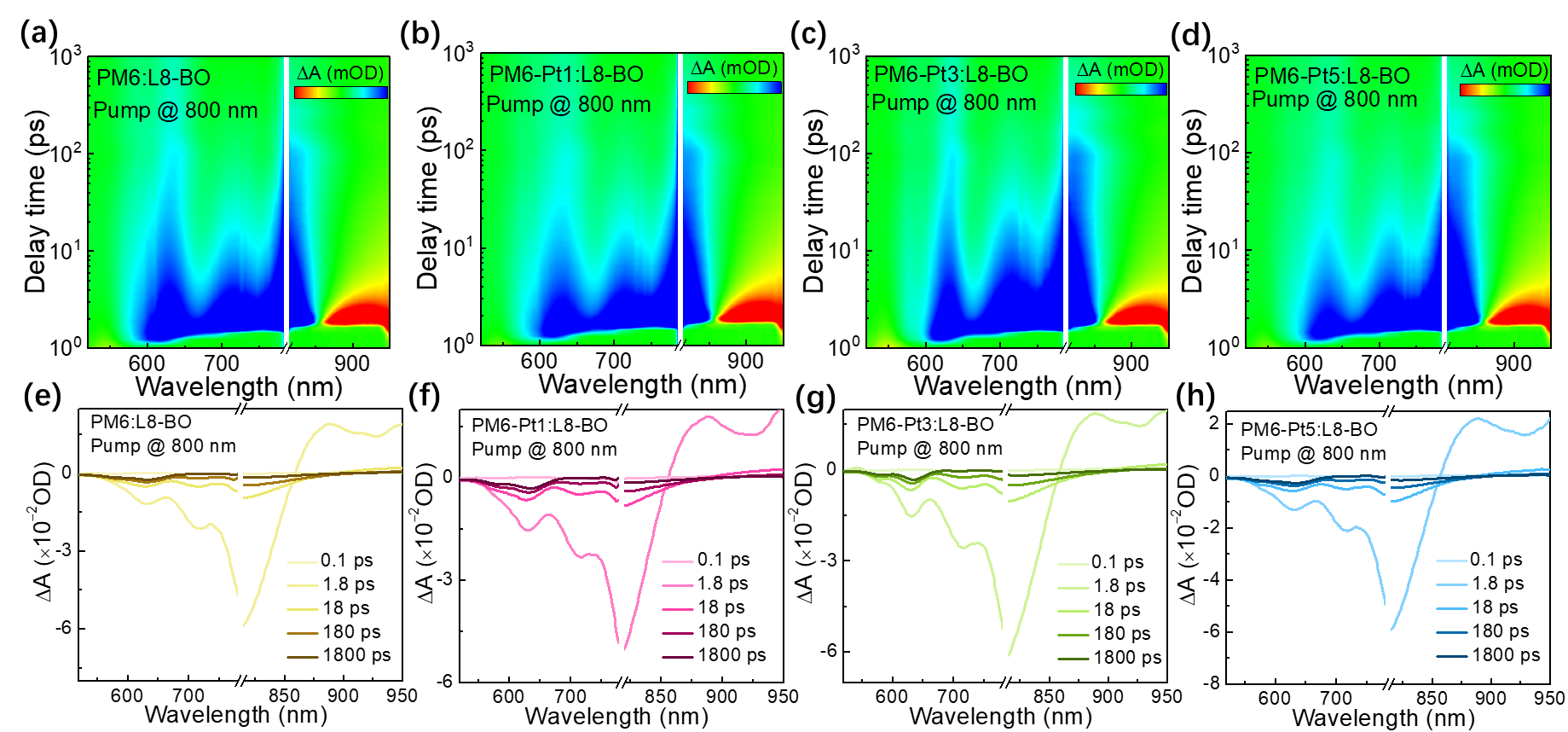
**

**Figure S24**. (a-d) Color plot of TA spectra of PM6:L8-BO, PM6-Pt1:L8-BO, PM6-Pt3:L8-BO and PM6-Pt5:L8-BO films under 800 nm excitation. (e-h) Representative TA spectra of PM6:L8-BO, PM6-Pt1:L8-BO, PM6-Pt3:L8-BO and PM6-Pt5:L8-BO films under 800 nm excitation.

**Figure S25**. TRPL decay spectra of PM6:L8-BO, PM6-Pt1:L8-BO, PM6-Pt3:L8-BO and PM6-Pt5:L8-BO blend films.

**Table S11** Detailed parameters of TRPL of the blend films were fitted by a biexponential function: *i* = A_1_ exp(−t/τ_1_) + A_2_ exp(−t/τ_2_), with two lifetimes of *τ*_1_ and *τ*_2_ and prefactors of *A*_1_ and *A*_2_.

| Blend films | *A*_1_ | *τ*_1_ (ps) | *A*_2_ | *τ*_2_ (ps) | *τ*_m_ (ps)^a)^ |
| --- | --- | --- | --- | --- | --- |
| PM6:L8-BO | 0.50 | 0.284 | 0.50 | 0.284 | 0.284 |
| PM6-Pt1:L8-BO | 0.50 | 0.249 | 0.50 | 0.249 | 0.249 |
| PM6-Pt3:L8-BO | 0.50 | 0.382 | 0.50 | 0.382 | 0.382 |
| PM6-Pt5:L8-BO | 0.50 | 0.402 | 0.50 | 0.402 | 0.402 |

^a)^ The average lifetime values were extracted by a biexponential function fit and calculated according to the equation: *τ*_m_ = (*A*_1_*τ*_1_^2^ + *A*_2_*τ*_2_^2^)/(*A*_1_*τ*_1_ + *A*_2_*τ*_2_)


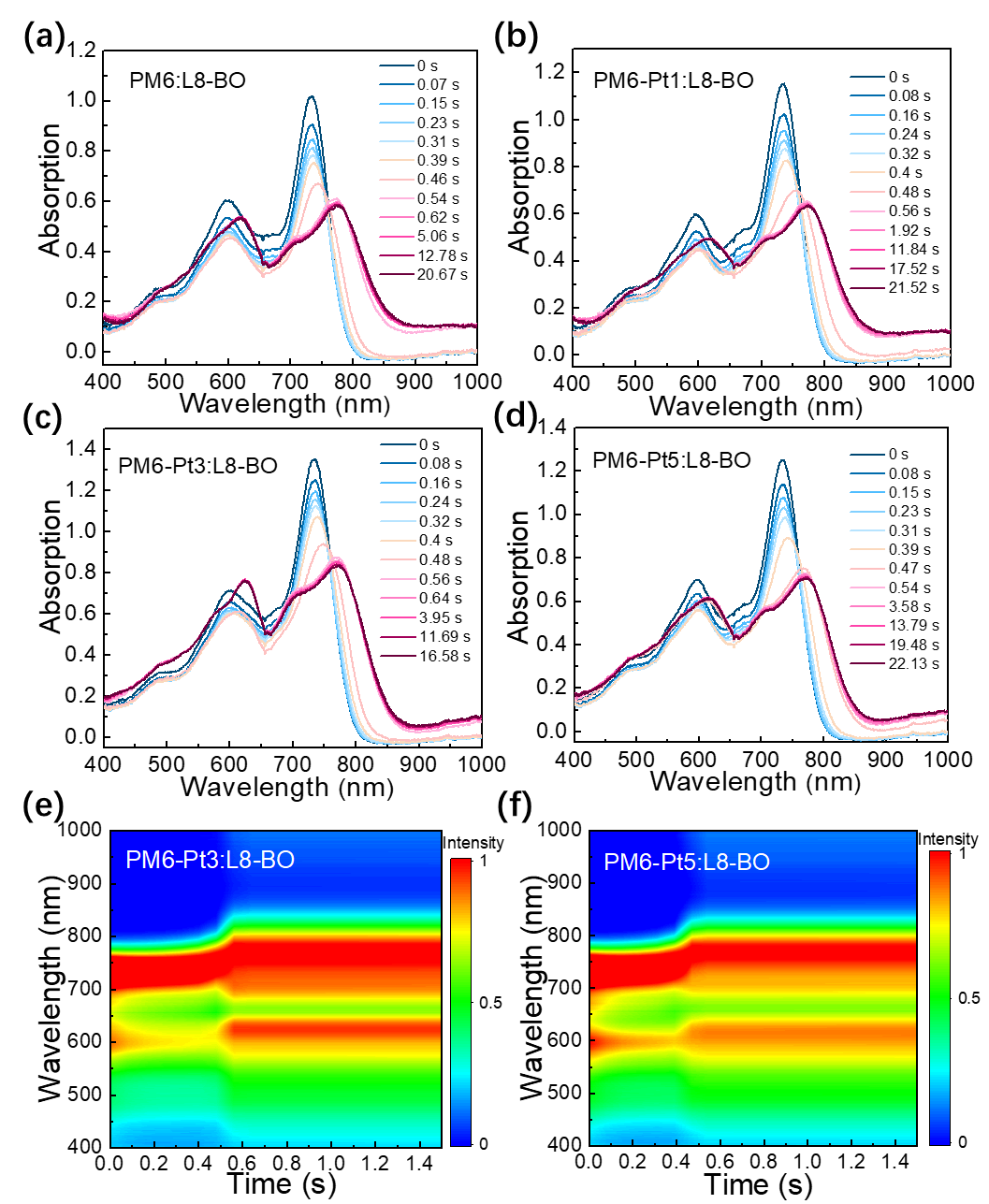


**Figure S26**. The in-situ UV–vis absorption spectra of (a) PM6:L8-BO, (b) PM6-Pt1:L8-BO, (c) PM6-Pt3:L8-BO and (d) PM6-Pt5:L8-BO blend films. Time-resolved UV-vis absorption spectra of (e) PM6-Pt3:L8-BO and (f) PM6-Pt5:L8-BO blend films.

**Figure S27**. The peak position evolution as a function of time for the blend films. Decay dynamics of excitons of polymer donors.


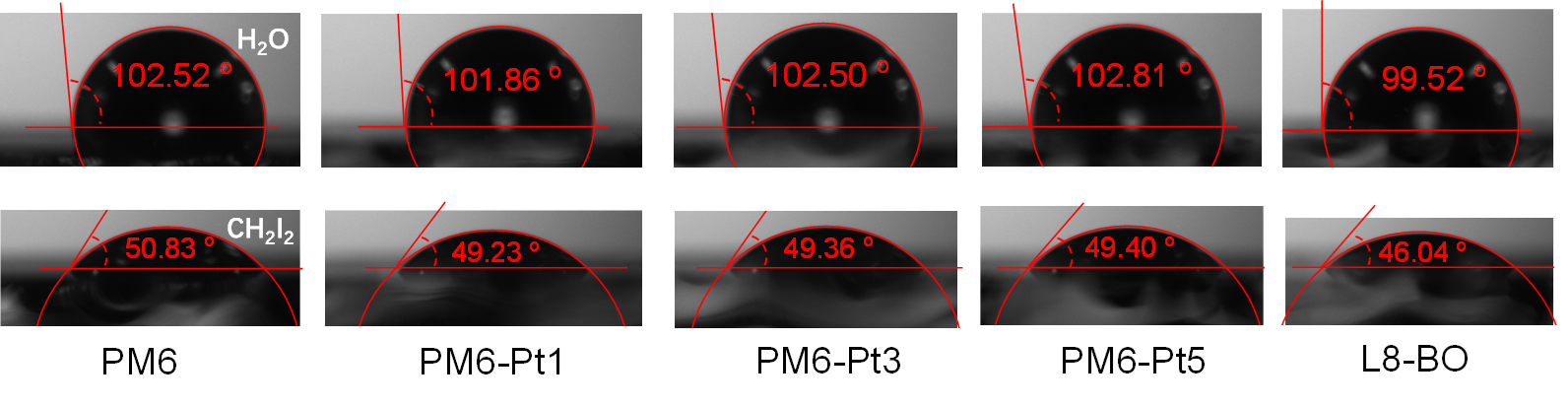


**Figure S28**. Contact angles of water and [diiodomethane](javascript:;) droplets on the PM6, PM6-Pt1, PM6-Pt3, PM6-Pt5 and L8-BO films.

**Table S12** The contact angles and surface tension parameters of the pure films.

| Pristine film | *θ*_water_  (deg) | *θ*_DIM_  (deg) | γ^d^  (mN/m) | γ^p^  (mN/m) | Surface tension  (mN/m) | χ |
| --- | --- | --- | --- | --- | --- | --- |
| PM6 | 102.52 | 50.83 | 34.13 | 0.97 | 35.10 | 0.033K |
| PM6-Pt1 | 101.86 | 49.23 | 34.78 | 1.08 | 35.86 | 0.014K |
| PM6-Pt3 | 102.50 | 49.36 | 35.08 | 0.83 | 35.91 | 0.013K |
| PM6-Pt5 | 102.81 | 49.40 | 35.28 | 0.7 | 35.98 | 0.012K |
| L8-BO | 99.52 | 46.04 | 35.58 | 1.73 | 37.31 | – |

γ^d^ and γ^p^ represent the surface free energy (γ) generated from the dispersion forces and the polar forces, respectively. Surface energy (γ) = γ^d^ + γ^p^


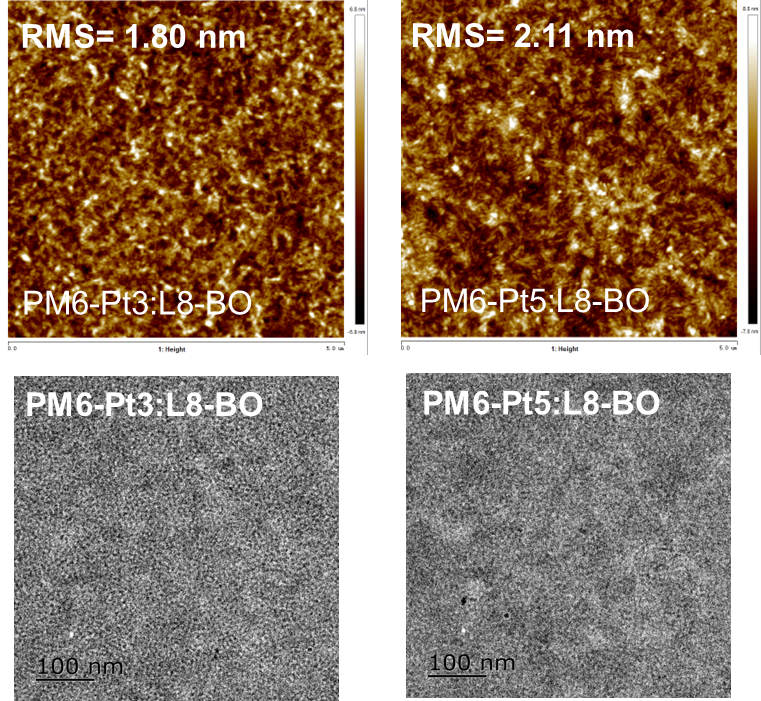


**Figure S29**. Tapping-mode AFM height images and TEM images of the corresponding binary blend films.


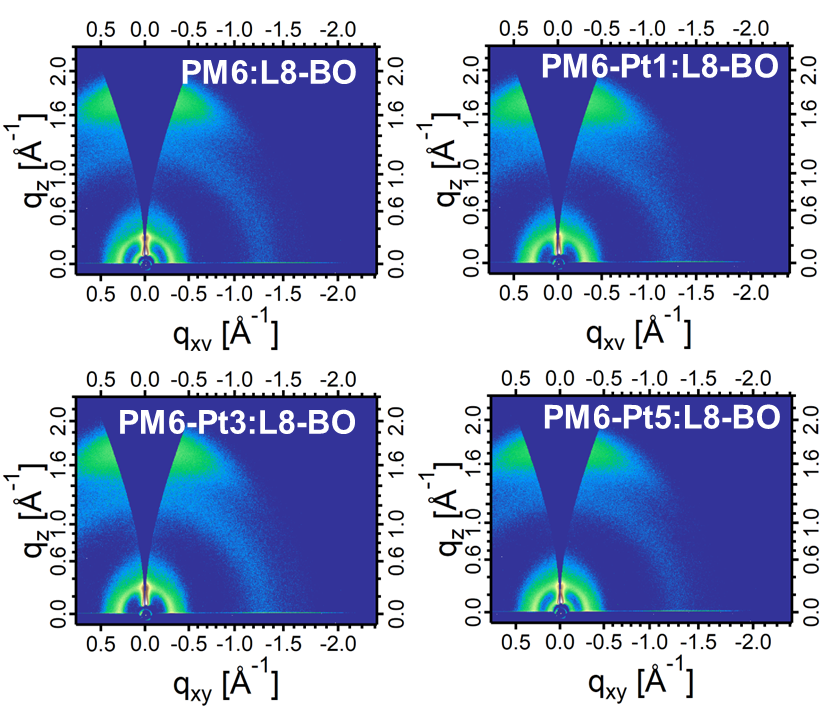


**Figure S30**. GIWAXS patterns of the blend films.

**Table S13** Morphology data of blend films.

| Film | in plane (IP) | | | | out of plane (OOP) | | | |
| --- | --- | --- | --- | --- | --- | --- | --- | --- |
|  | Position (Å^-1^) | d-spacing*^a^*  Å | FWHM  Å | CCL*^b^*  Å | Position (Å^-1^) | d-spacing*^a^*  Å | FWHM  Å | CCL*^b^*  Å |
| PM6:L8-BO | 0.304 | 20.65 | 0.068 | 83.11 | 1.73 | 3.63 | 0.291 | 19.42 |
| PM6-Pt1:L8-BO | 0.300 | 20.93 | 0.076 | 74.36 | 1.73 | 3.63 | 0.306 | 18.47 |
| PM6-Pt3:L8-BO | 0.304 | 20.65 | 0.081 | 69.77 | 1.73 | 3.63 | 0.335 | 16.87 |
| PM6-Pt5:L8-BO | 0.306 | 20.52 | 0.099 | 57.09 | 1.73 | 3.63 | 0.348 | 16.24 |

*^a^* Obtained using the equation of *d* = 2π/*q*, in which *q* is the corresponding *x*-coordinate of the diffraction peak. *^b^* Calculated using the equation: CCL= 2π*K*/*w*, in which *w* is the full width at half maximum and *K* is a form factor.


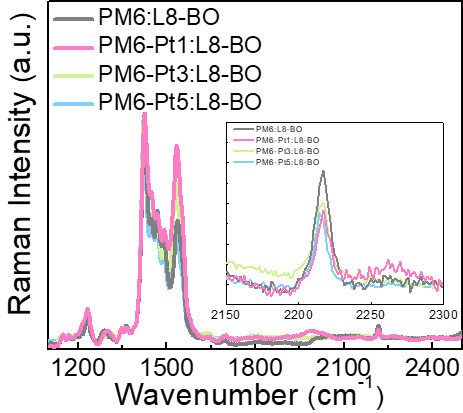


**Figure S31**. The Raman spectra of PM6:L8-BO, PM6-Pt1:L8-BO, PM6-Pt3:L8-BO and PM6-Pt5:L8-BO blend films.


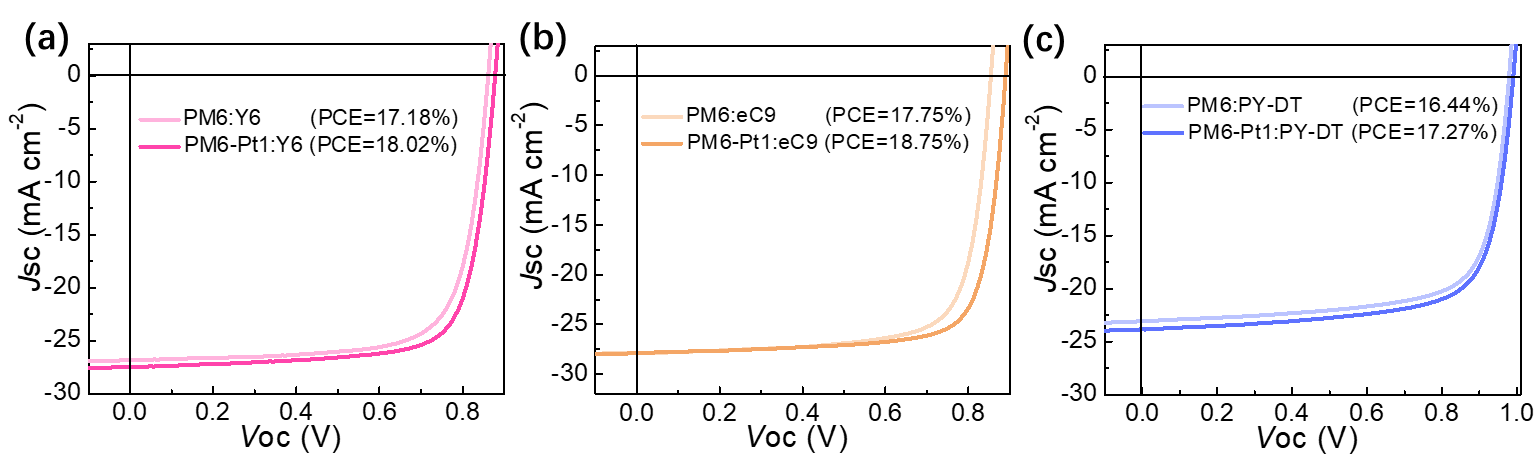


**Figure S32**. *J*−*V* characteristics of the OSCs based on Y6, eC9 and PY-DT as electron acceptors under AM1.5G illumination (100 mW cm^−2^).


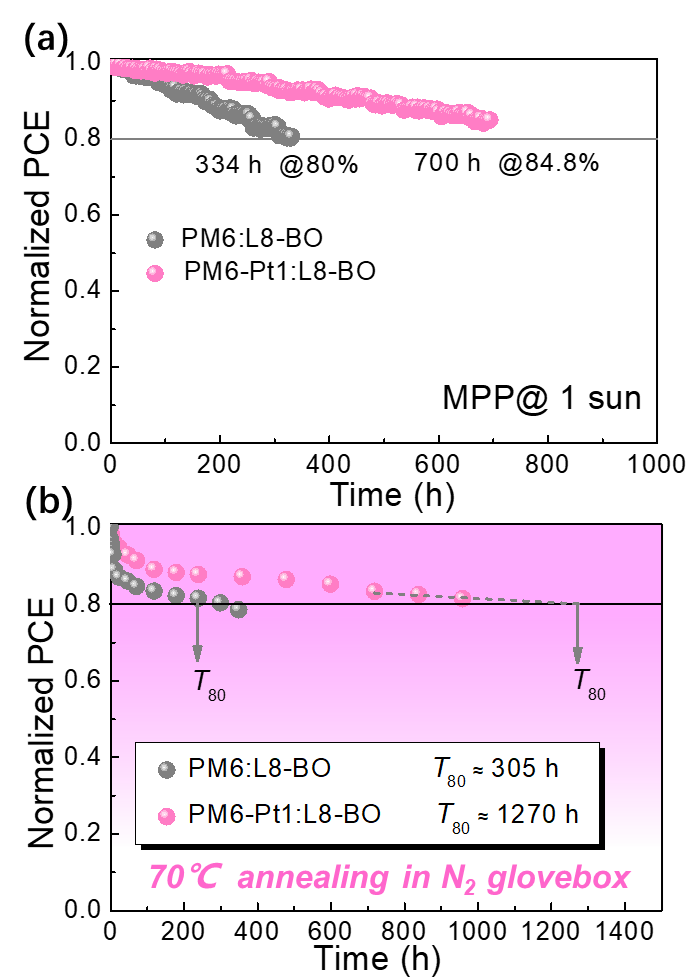


**Figure S33**. (a) MPP tracking for PM6:L8-BO and PM6-Pt1:L8-BO devices measured under 1 sun illumination. (b) Thermal stability for inverted-structure devices based on PM6:L8-BO and PM6-Pt1:L8-BO under 70 ℃ continuous annealing in a N_2_ glovebox.

**1.15 ^1^H NMR, ^13^C NMR and HRMS Spectra of the intermediate compounds**


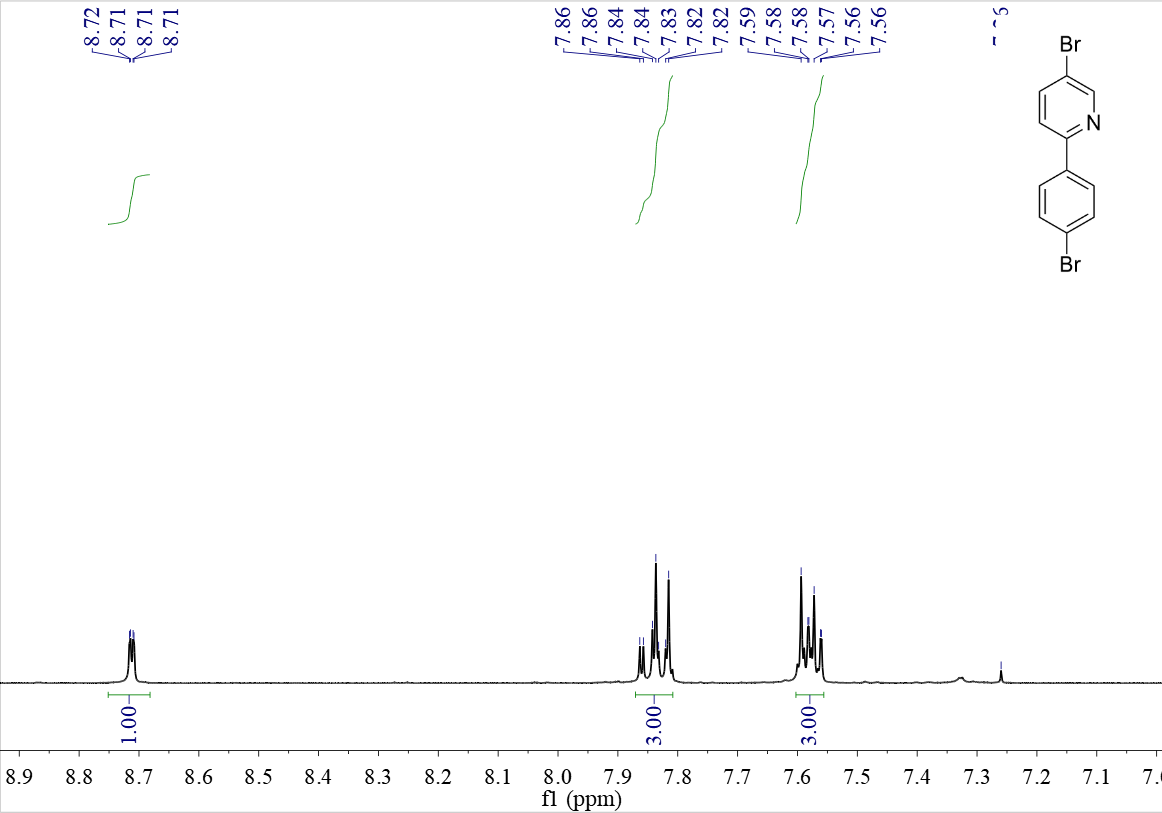


**Figure S34.** ^1^H NMR spectrum of compound **1** (CDCl_3_, 400 MHz).


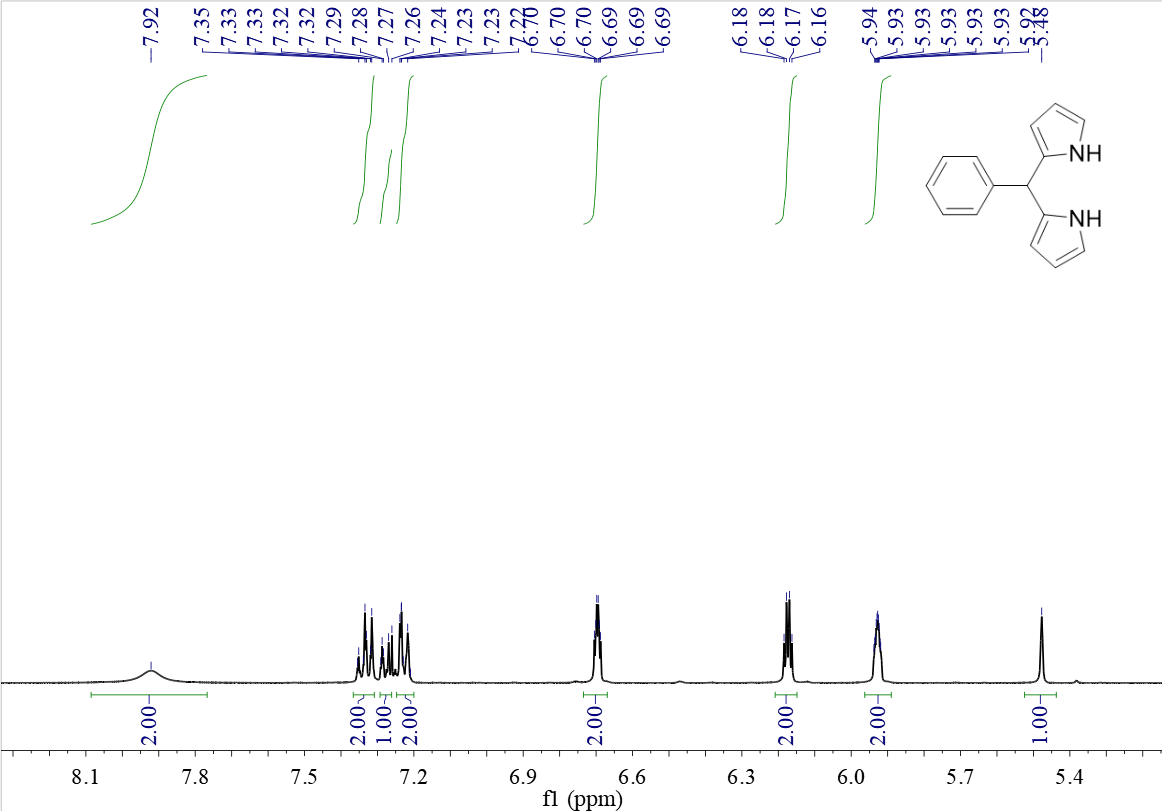


**Figure S35.** ^1^H NMR spectrum of compound **3** (CDCl_3_, 400 MHz).


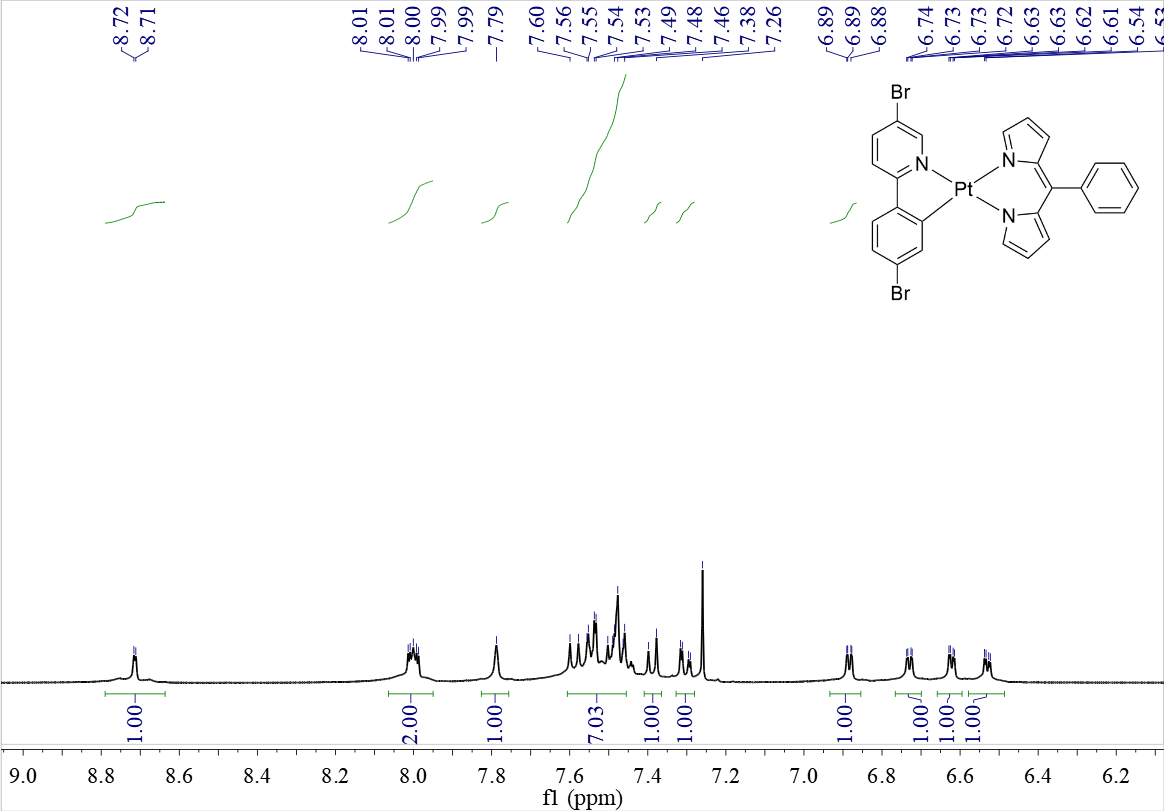


**Figure S36.** ^1^H NMR spectrum of compound **5** (CDCl_3_, 400 MHz).


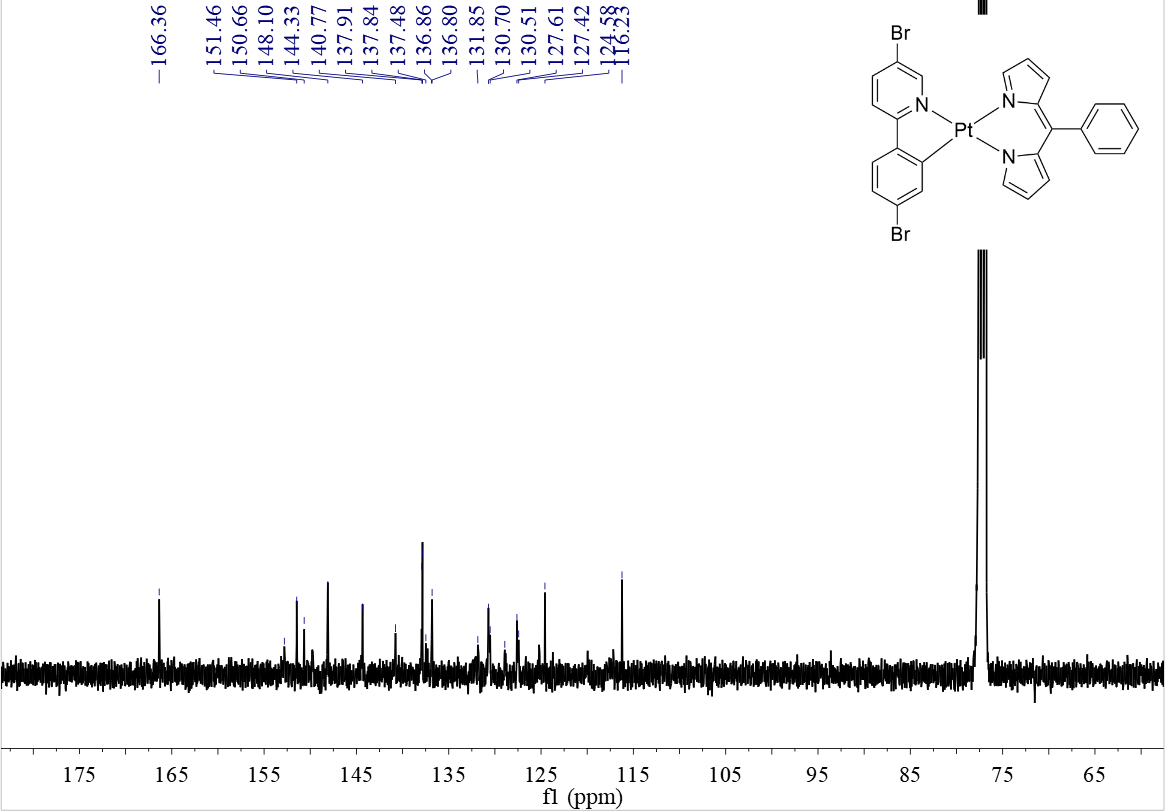


**Figure S37.** ^13^C NMR spectrum of compound **5** (CDCl_3_, 400 MHz).


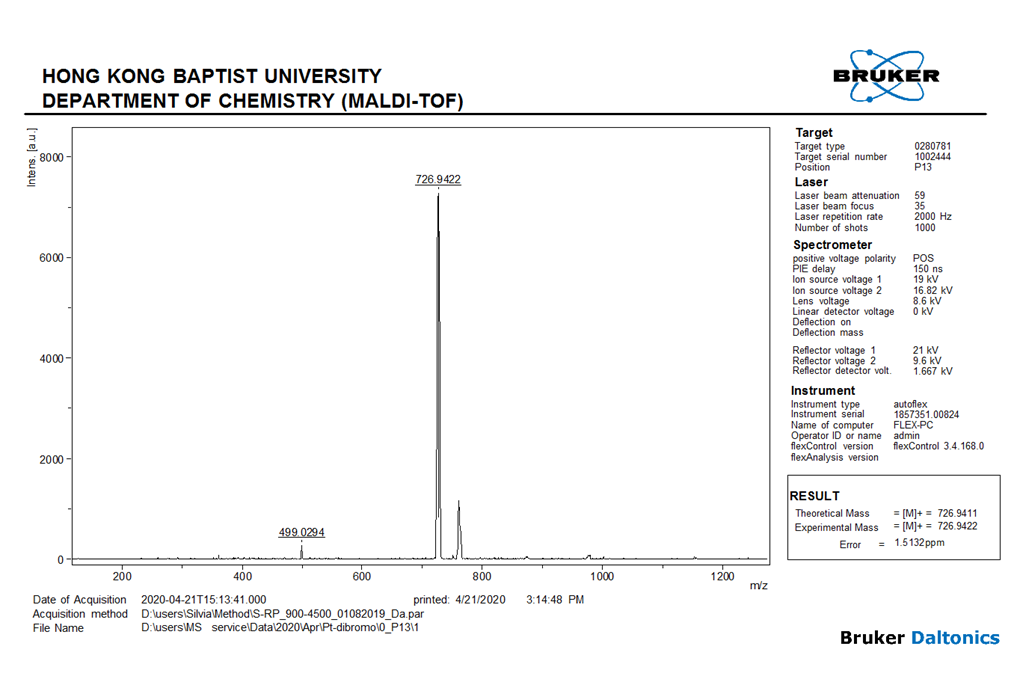


**Figure S38.** MALDI-TOF mass spectrum of compound 5.

References

[1] G. G. Malliaras, J. R. Salem, P. J. Brock, C. Scott, *Phys. Rev. B.* **1998**, *58*, 13411.

[2] T. Hua, J. Miao, H. Xia, Z. Huang, X. Cao, N. Li, C. Yang, *Adv. Funct. Mater.* **2022**, *32*, 2201032.

[3] K.-N. Zhang, X.-Y. Du, Z.-H. Chen, T. Wang, Z.-Q. Yang, H. Yin, Y. Yang, W. Qin, X.-T. Hao, *Adv*. *Energy Mater*. **2022**, *12*, 2103371.

[4] S. Chandrabose, K. Chen, A. J. Barker, J. J. Sutton, S. K. K. Prasad, J. Zhu, J. Zhou, K. C. Gordon, Z. Xie, X. Zhan, J. M. Hodgkiss, *J. Am. Chem. Soc.* **2019**, *141*, 6922-6929.

[5] D. Luo, L. Li, E. Zhou, W.-Y. Wong, A. K. K. Kyaw, *Mater. Adv.* **2023**, *4*, 4444.
